# Revisiting the scorpion central nervous system using microCT

**DOI:** 10.1038/s41598-024-76917-6

**Published:** 2024-11-14

**Authors:** Stephanie F. Loria, Valentin L. Ehrenthal, Lauren A. Esposito

**Affiliations:** 1 Institute for Biodiversity Science and Sustainability, California Academy of Sciences, San Francisco, CA USA; 2 Museum of Nature Hamburg-Zoology, Leibniz Institute for the Analysis of Biodiversity Change, Hamburg, Germany; 3 Department of Biology, University of Hamburg, Hamburg, Germany

**Keywords:** Scorpions, Central nervous system, Brain, Prosomal ganglion, Arcuate body, Mushroom body, Evolution, Neuroscience, Zoology

## Abstract

The central nervous system (CNS) of Chelicerata has remained conserved since the Cambrian, yet few studies have examined its variability within chelicerate orders including Scorpiones. The scorpion CNS comprises the prosomal ganglion and opisthosomal ventral nerve cord. We visualize the scorpion CNS with microCT, explore morphological variation across taxa, compare the scorpion CNS to other arachnids, and create a terminology glossary and literature review to assist future studies. Six scorpion species were microCT scanned. Scan quality varied and most structures in the prosomal ganglion could only be observed in *Paruroctonus becki* (Vaejovidae). Major nerves and the first opisthosomal ganglion were visible in nearly all taxa. We present the most detailed 3D-rendering of the scorpion prosomal ganglion to date. Our results corroborate existing research and find the scorpion CNS to be conserved. Nearly all structures reported previously in the prosomal ganglion were located in similar positions in *P. becki*, and nerve morphology was conserved across examined families. Despite similarities, we report differences from the literature, observe taxonomic variation in prosomal ganglion shape, and confirm positional variation for the first opisthosomal ganglion. This study serves as a starting point for microCT analysis of the scorpion CNS, and future work should include more distantly related, size variable taxa to better elucidate these findings.

**Supplementary Information:**

The online version contains supplementary material available at 10.1038/s41598-024-76917-6.

## Introduction

The central nervous system (CNS) of many panarthropod groups is highly conserved^1^. Comparison of well-preserved, Cambrian panarthropod fossils with their respective extant relatives in Myriapoda, Pancrustacea and Onychophora, and with histological and phylogenetic data, suggests that distinct neural ground plans for each Panarthropoda subphylum evolved between the Ediacaran and lower Cambrian and have remained relatively unchanged since. For the subphylum Chelicerata, the extinct megacheiran genus *Alalcomenaeus* Simonetta, 1970 (Family Leanchoiliidae Raymond, 1935), a putative stem lineage, demonstrates that the chelicerate neural ground plan—in which only the first-order visual neuropils of each eye are positioned separately from the rest of the brain^1^—dates to at least 518 million years ago, but was probably laid down even earlier^1,2^. Although the chelicerate CNS is constrained by this ancient blueprint, few studies have assessed morphological variability of the CNS within chelicerate groups or have tried to understand its evolution. Among sea spiders (Class Pycnogonida), microCT data indicates that evolution of the CNS corresponds with changes to external morphology, and convergent evolution has played an important role in its gross layout^3^.

The chelicerate order Scorpiones is a mesodiverse (~ 2800 sp.)^4^ lineage in the Class Arachnida with the earliest fossils dating to the Silurian. Interest in scorpion CNS morphology took off in the middle of the nineteenth century, but most research has focused on its structure in individual species using histology (Table S1). The seminal work by Babu^5^ remains the reference study on scorpion neuroanatomy, providing detailed descriptions and illustrations of the CNS, and summarizing previous literature. Since then, most studies on scorpion internal anatomy using more advanced techniques have only tangentially investigated the CNS, usually as a secondary outcome following examination of morphological characters adjacent to it. Considering all morphological research on the scorpion CNS, it appears to be highly conserved across taxa. However, to date, no studies have applied microCT data to explore morphological variation in the scorpion CNS or use it as a means to observe neuroanatomical characters in more detail, as has been done in other arachnid taxa (e.g., spiders^6^, camel spiders^7^). Moreover, terminology in scorpion neuroanatomy varies throughout the literature and has not been standardized, and our understanding of homologies of neuroanatomical characters across arthropods is limited, making comparison of the scorpion CNS with other arachnids difficult. We borrow neuroanatomical terms from other arthropods and apply them to scorpions throughout, but caution the reader that a thorough assessment of the CNS across Arthropoda is essential to evaluate if these structures are indeed homologous. A further point of clarification– our definitions of scorpion CNS structures may differ from those applied in other scorpion neuroanatomical papers, however, we provide a glossary to assist with interpretation (Table S2).

Here, we review past research on the scorpion CNS, and present the most detailed rendering of the scorpion prosomal ganglion and its component structures (e.g., arcuate body, mushroom bodies, optic neuropils) to date using microCT. We also compare the scorpion CNS with that of other arachnids and explore morphological variation across scorpion taxa.

### A review of the scorpion central nervous system

The scorpion CNS refers to the prosomal ganglion and opisthosomal ventral nerve cord^5,8,9^. The prosomal ganglion comprises the brain, subesophageal mass, circumesophageal commissures and associated nerves^8,9^. It is situated under the scorpion carapace and is pierced by the esophagus^5,10,11^. The definition of ‘brain’ across Arthropoda is a complicated and often contested topic (see, for example, Scholtz & Edgecombe^12^, Bitsch and Bitsch^13^ or Richter et al.^14^ for a discussion on the arthropod head and brain with references therein, and Wolf^15^ on the term ‘brain’ in scorpions). However, for the present study, we consider the scorpion brain to comprise the protocerebrum and deutocerebrum, whereas the tritocerebrum is part of the subesophageal mass^16^. The protocerebrum is situated dorso-anteriorly and includes the optic neuropils, paired mushroom bodies, which connect to the lateral eyes in other arachnids^6,17^, and the arcuate body, which connects to the median eyes in scorpions^18^. The deutocerebrum, which innervates the first antennae in Mandibulata, was previously thought to be absent or reduced in Chelicerata^5,13,19^, however, genetic and developmental research indicate that it is present and innervates the cheliceral neuropil^10,20–22^. The tritocerebrum has been identified as the pedipalpal neuropil in arachnids (spiders^6^, scorpions^11,16^); we follow Klußmann-Fricke^16^ for its placement in the subesophageal mass of scorpions. The subesophageal mass, situated ventral to the brain, consists of multiple fused ganglia with their component neuropils (see Richter et al.^10^: fig. 9 for structure of a ganglion), and is connected to the brain by short circumesophagael commissures. The number of fused ganglia in the subesophageal mass varies in the literature (e.g.,^23,24^) but we recognize the following: one pedipalpal ganglion; four pedal ganglia for legs I–IV; four mesosomal ganglia that have been incorporated into the subesophageal mass including pectinal, genital pore and book lung ganglia; and the central ganglion^16,23,25^. The subesophageal mass also includes longitudinal fibre and other connecting tracts, transverse commissures and the ventral association center^5^. Shielding the prosomal ganglion are the endosternite, gnathobases and the neurilemma^5,16,26^—a tissue that is especially thickened between the sternum and subesophageal mass in arachnids^26^. A large capillary network, referred to as the bipolar *rete mirabile*, transports hemolymph in and out of the prosomal ganglion^16,26,27^.

Posterior to the prosomal ganglion is the opisthosomal ventral nerve cord. It includes seven free opisthosomal ganglia (OG1–OG7), three in the mesosoma and four in the metasoma, and their associated nerves^8^. Ganglia in the mesosomal segments of the opisthosoma (O1–O8) innervate the book lungs, but their positions vary across taxa^8,28–31^: OG1 is situated between sternites I and II (O4–O5); OG2 between sternites III and IV (O6–O7); and OG3 in sternite V (O8). Ganglia positions are more stable in the metasomal segments of the opisthosoma (O9–O13): OG4 in metasomal segment I (O9), OG5 between metasomal segments I and II (O9–O10), OG6 between metasomal segments II and III (O10–O11), and OG7 between metasomal segments III and IV (O11–O12). A pair of branching lateral nerves stem from each ganglion and innervate muscles and integument on the dorsal and ventral surfaces^5,8^. Interestingly, several additional nerve pairs originate from OG7—a composite of two fused ganglia—including the telsonic, metasomal segment V and alimentary nerves. The telsonic and mestasomal segment V nerves are larger than the lateral nerves, stem posteriorly from OG7, and innervate muscles in the telson and metasomal segment V, respectively. In contrast, alimentary nerves are smaller, originate postero-dorsally from OG7 and innervate the digestive tract^5,8^.

## Results

### Scan quality

**Fig. 1:**
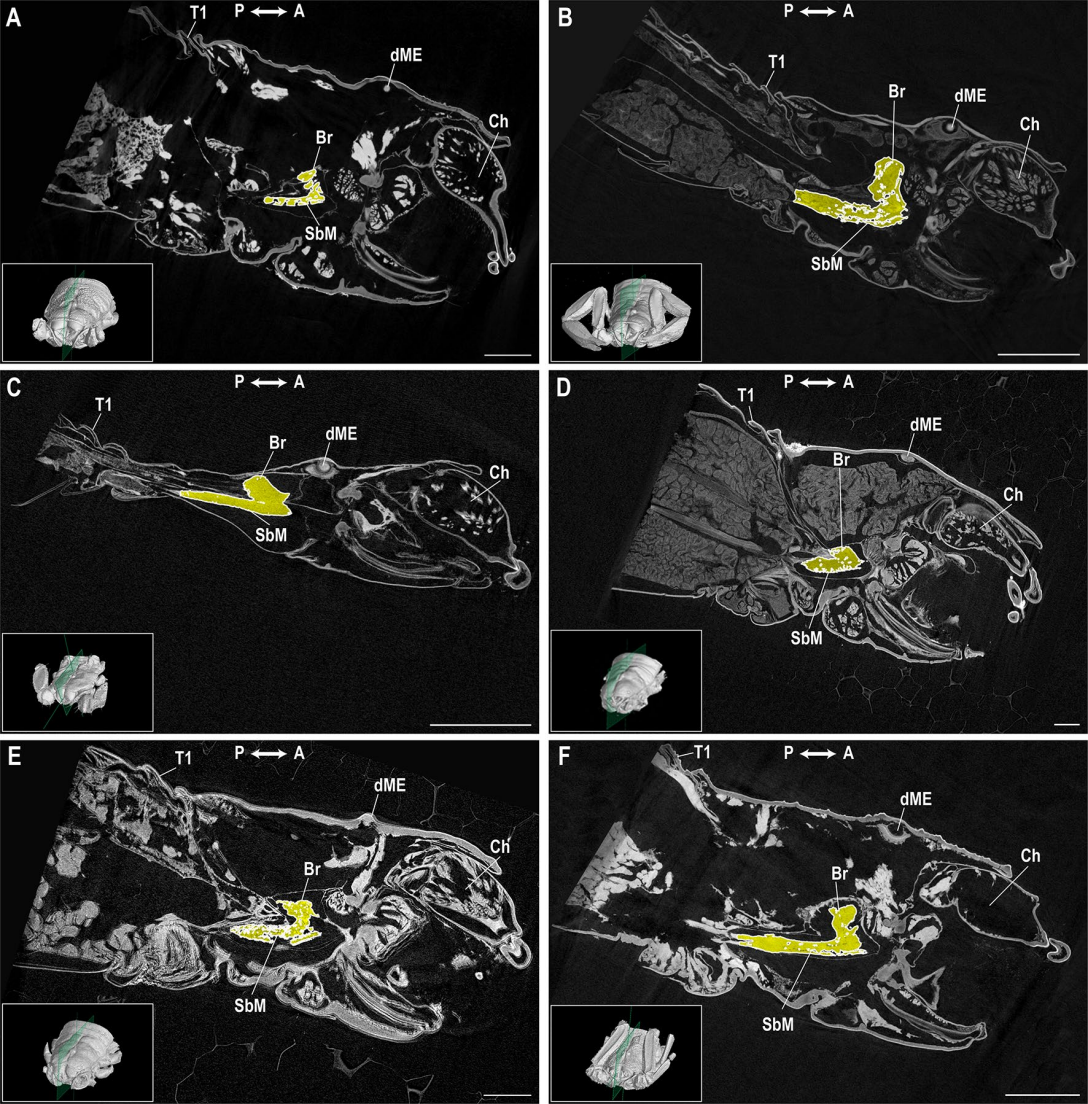
Parasagittal view of prosomal ganglion. Parasagittal view, anterior-posterior axis of the prosomal ganglion in Vaejovidae Thorell, 1876 (**A**,** B**), Hormuridae Laurie, 1896 (**C**), Hadruridae Stahnke, 1974 (**D**), Chactidae Pocock, 1893 (**E**), and Buthidae C.L. Koch, 1837 (**F**). (**A**) *Paravaejovis spinigerus* (Wood, 1863); (**B**) *Paruroctonus becki* (Gertsch & Allred, 1965); (**C**) *Liocheles australasiae* (Fabricius, 1775); (**D**) *Hadrurus arizonensis* Ewing, 1928; (**E**) *Uroctonus mordax* Thorell, 1876; (**F**) *Centruroides sculpturatus* Ewing, 1928. Insets indicate the parasagittal plane in the prosoma for each species.* Br* brain,* Ch* chelicera,* dME* dextral median eye,* SbM* subesophageal mass,* T1* first tergite. Axis indicator abbreviations:* A* anterior,* P* posterior. Scale bars = 1 mm.

**Fig. 2:**
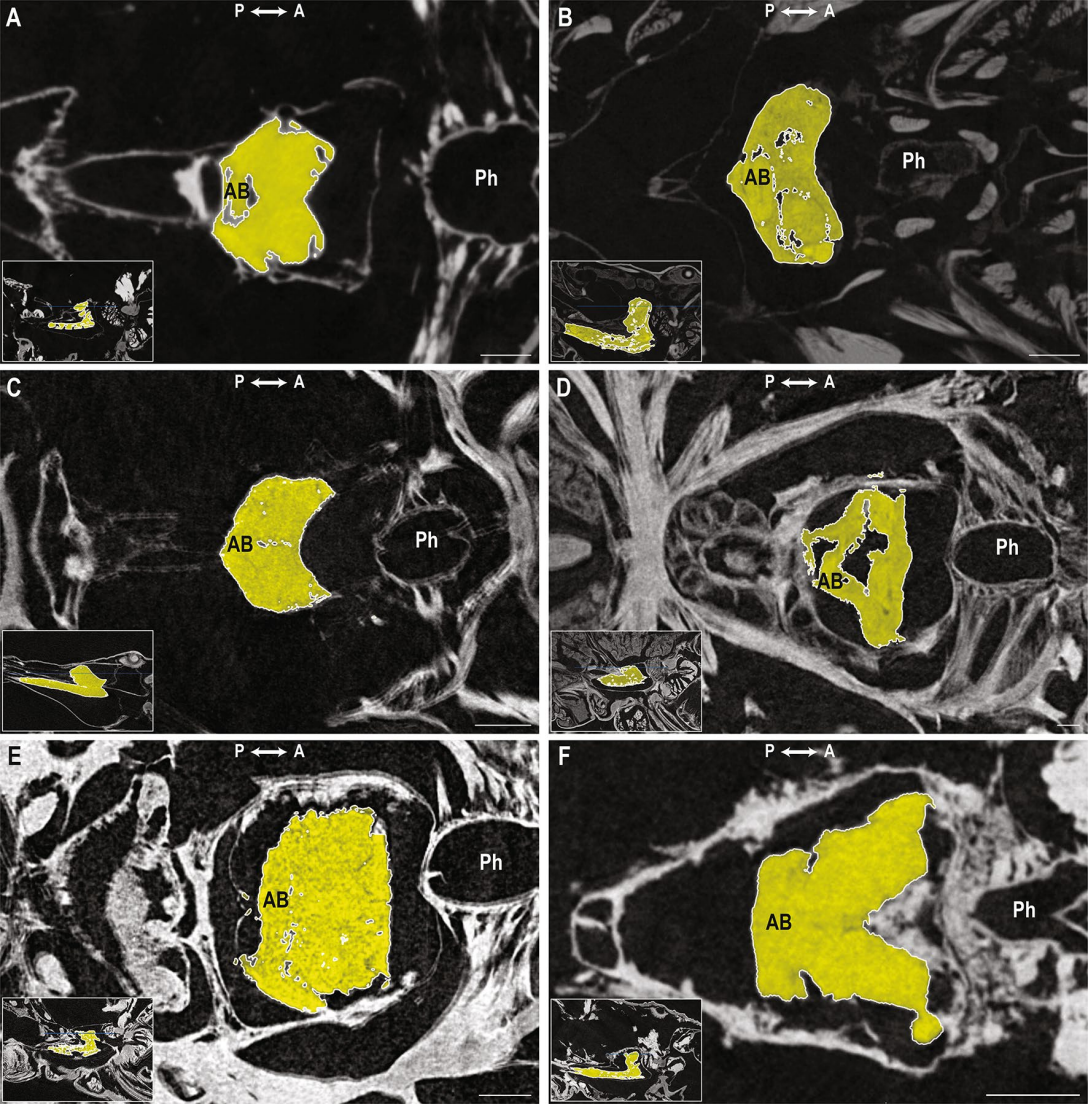
Transverse view of the prosomal ganglion. Transverse view, dorsal-ventral axis of the prosomal ganglion in Vaejovidae Thorell, 1876 (**A**,** B**), Hormuridae Laurie, 1896 (**C**), Hadruridae Stahnke, 1974 (**D**), Chactidae Pocock, 1893 (**E**), and Buthidae C.L. Koch, 1837 (**F**). (**A**) *Paravaejovis spinigerus* (Wood, 1863); (**B**) *Paruroctonus becki* (Gertsch & Allred, 1965); (**C**) *Liocheles australasiae* (Fabricius, 1775); (**D**) *Hadrurus arizonensis* Ewing, 1928; (**E**) *Uroctonus mordax* Thorell, 1876; (**F**) *Centruroides sculpturatus* Ewing, 1928. Insets indicate the transverse plane in the prosoma for each species.* AB* arcuate body,* Ph* pharynx. Axis indicator abbreviations:* A* anterior,* P* posterior. Scale bars = 0.2 mm.

Scan quality varied across taxa (Tables S3, S4; Figs. 1 and 2). Resolution among prosomal scans was ~ 3–4 μm for *Paruroctonus becki* (Gertsch & Allred, 1965), *Liocheles australasiae* (Fabricius, 1775) and *Centruroides sculpturatus* Ewing, 1928; ~ 6.7 μm for *Paravaejovis spinigerus* (Wood, 1863) and *Uroctonus mordax* Thorell, 1876; and ~ 10.9 μm for *Hadrurus arizonensis* Ewing, 1928 (Table S4). Resolution was lowest for the full body scan of *P. becki* (voxel size ≥ 50 μm). Tissue contrast also differed across scans. Contrast was best in the prosomal scan of *P. becki*, and also high in *H. arizonensis*, but low in all other scans. A large air gap was observed within the brain of *H. arizonensis*, suggesting damage occurred during sample preparation (Fig. 2D). A motion artifact was detected in *U. mordax*.

### Prosomal ganglion: brain

**Fig. 3:**
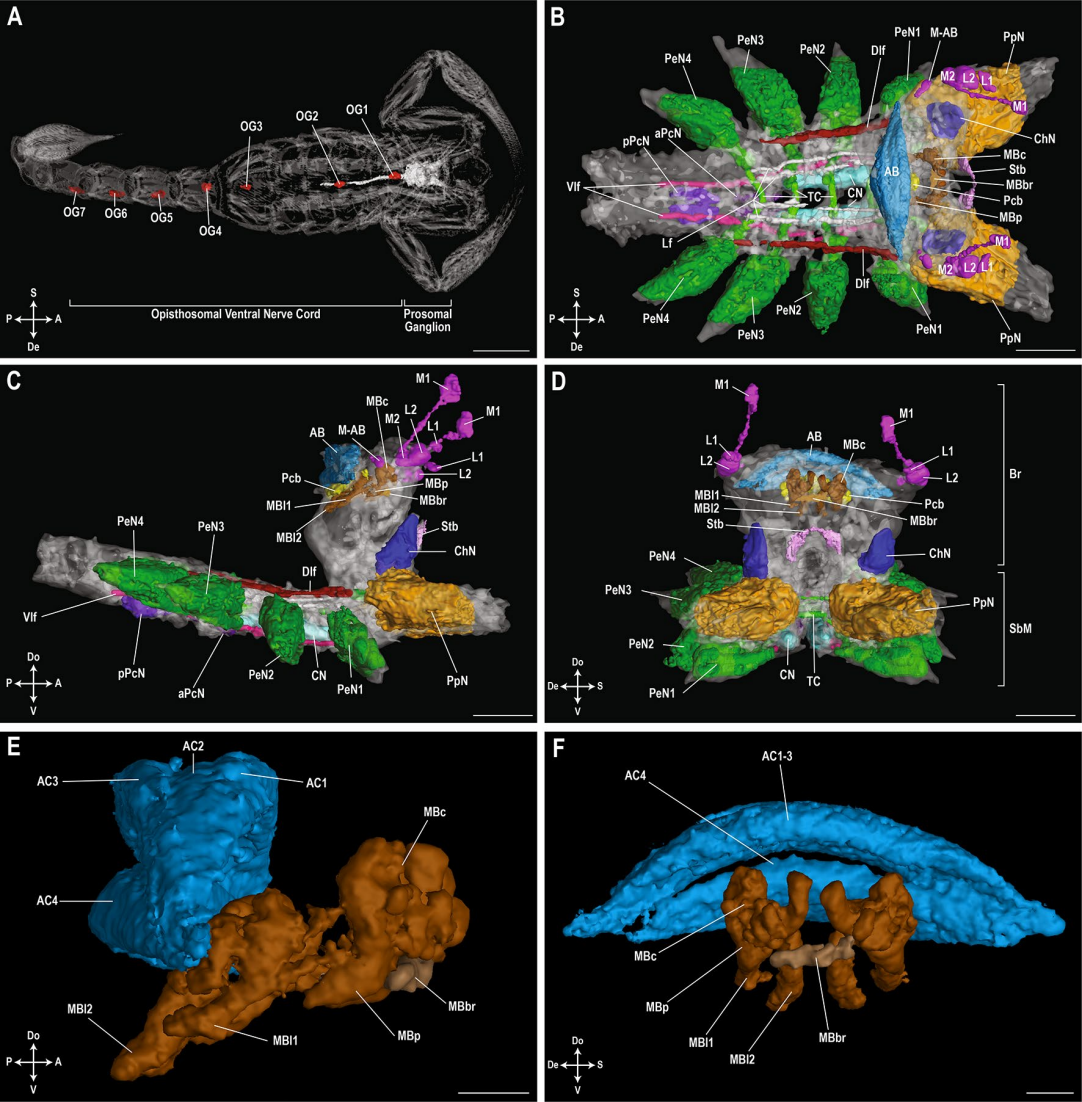
Central nervous system of *Paruroctonus becki* (Gertsch & Allred, 1965). 3D-renderings of the central nervous system (CNS) of *P. becki* in dorsal (**A**, **B**), lateral (**C**, **E**) and frontal views (**D**, **F**), including overview of CNS with the ganglia of the opisthosomal ventral nerve cord (**A**), prosomal ganglion (**B–D**) and mushroom body with arcuate body (**E**, **F**).* AB* arcuate body,* AC1–AC4* four arcuate body lobes,* aPcN* anterior pectinal neuropil,* Br* brain,* ChN* cheliceral neuropil,* CN* central neuropil,* Dlf* dorso-lateral fibre tract,* L1* first-order lateral eye neuropil,* L2* second-order lateral eye neuropil,* Lf* longitudinal fibre tract,* M1* first-order median eye neuropil,* M2* second-order median eye neuropil,* MBbr* mushroom body bridge,* MBc* mushroom body calyx,* MBp* mushroom body pedunculus,* MBl1–MBl2* first and second mushroom body lobes,* M-AB* tract interconnecting the median eye neuropils with the arcuate body,* OG1–OG7* opisthosomal ganglia 1–7,* PeN1–PeN4* pedal neuropils for legs I–IV,* Pcb* protocerebral bridge,* pPcN* posterior pectinal neuropil,* PpN* pedipalpal neuropil,* SbM* subesophageal mass,* Stb* stomodeal bridge,* TC* transverse commissures,* Vlf* ventro-lateral fibre tract. Axis indicator abbreviations:* A* anterior,* De* dextral,* Do* dorsal,* P* posterior,* S* sinistral,* V* ventral. Scale bars: (**A**) = 2 mm; (**B**–**D**) = 0.2 mm; (**E**, **F**) = 0.05 mm. 3D-renderings available in MorphoSource (https://www.morphosource.org; Project ID:  000592858; DOIs: 10.17602/M2/M593451; 10.17602/M2/M592979; 10.17602/M2/M592982).

**Fig. 4:**
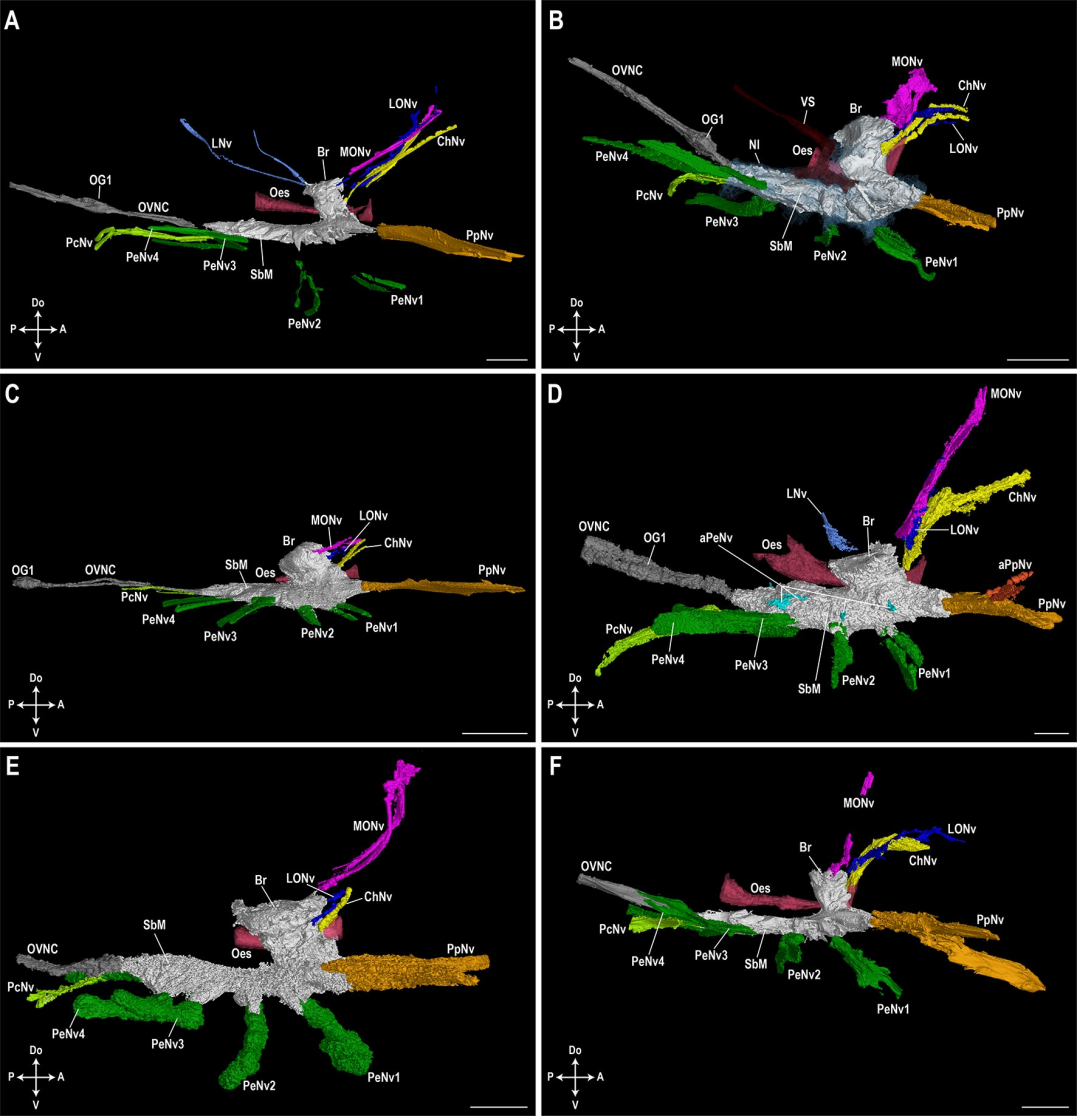
Lateral view of prosomal ganglion and associated nerves. 3D-renderings of the prosomal ganglion and associated nerves in lateral view in Vaejovidae Thorell, 1876 (**A**, **B**), Hormuridae Laurie, 1896 (**C**), Hadruridae Stahnke, 1974 (**D**), Chactidae Pocock, 1893 (**E**), and Buthidae C.L. Koch, 1837 (**F**). (**A**) *Paravaejovis spinigerus* (Wood, 1863); (**B**) *Paruroctonus becki* (Gertsch & Allred, 1965); (**C**) *Liocheles australasiae* (Fabricius, 1775); (**D**) *Hadrurus arizonensis* Ewing, 1928; (**E**) *Uroctonus mordax* Thorell, 1876; (**F**) *Centruroides sculpturatus* Ewing, 1928.* aPeNv* accessory pedal nerves,* aPpNv* accessory pedipalpal nerves,* Br* brain,* ChNv* cheliceral nerves,* LNv* lateral nerves,* LONv* lateral ocular nerves,* MONv* median ocular nerves,* Nl* neurilemma,* Oes* esophagus,* OG1* first opisthosomal ganglion, *OVNC* opisthosomal ventral nerve cord,* PcNv* pectinal nerves,* PeNv 1–4* pedal nerves for legs I–IV,* PpNv* pedipalpal nerves,* SbM* subesophageal mass,* VS* vascular system. Axis indicator abbreviations:* A* anterior,* Do* dorsal,* P* posterior,* V* ventral. Scale bars = 0.5 mm. 3D-renderings available in MorphoSource (https://www.morphosource.org; Project ID: 000592858; DOIs: 10.17602/M2/M593464; 10.17602/M2/M592973; 10.17602/M2/M592970; 10.17602/M2/M592967; 10.17602/M2/M593461; 10.17602/M2/M592964).

**Fig. 5:**
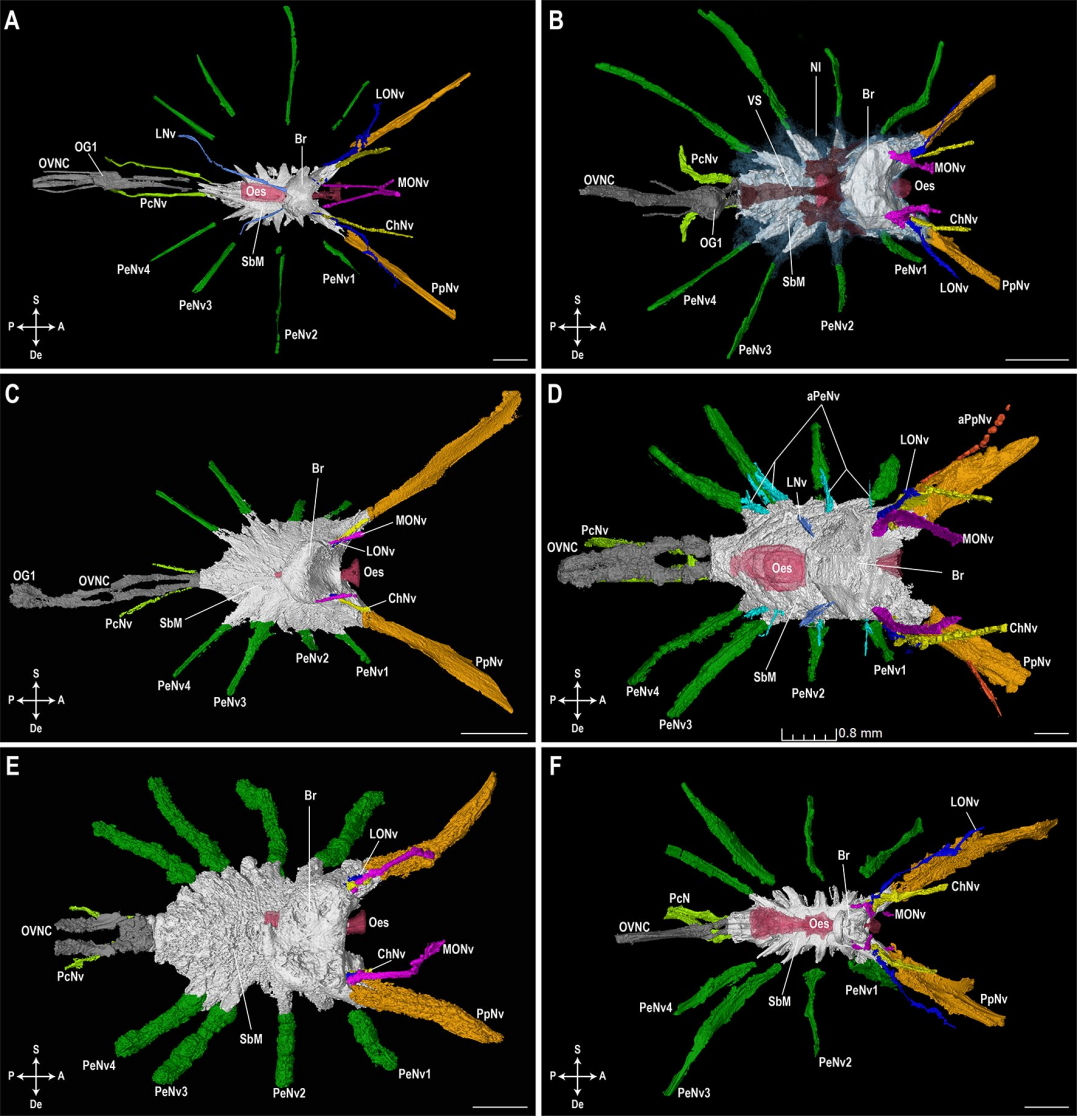
Dorsal view of prosomal ganglion and associated nerves. 3D-renderings of the prosomal ganglion and associated nerves in dorsal view in Vaejovidae Thorell, 1876 (**A**,** B**), Hormuridae Laurie, 1896 (**C**), Hadruridae Stahnke, 1974 (**D**), Chactidae Pocock, 1893 (**E**), and Buthidae C.L. Koch, 1837 (**F**). (**A**) *Paravaejovis spinigerus* (Wood, 1863); (**B**) *Paruroctonus becki* (Gertsch & Allred, 1965); (**C**) *Liocheles australasiae* (Fabricius, 1775); (**D**) *Hadrurus arizonensis* Ewing, 1928; (**E**) *Uroctonus mordax* Thorell, 1876; (**F**) *Centruroides sculpturatus* Ewing, 1928.* aPeNv* accessory pedal nerves,* aPpNv* accessory pedipalpal nerves,* Br* brain,* ChNv* cheliceral nerves,* LNv* lateral nerves,* LONv* lateral ocular nerves,* MONv* median ocular nerves,* Nl* neurilemma,* Oes* esophagus,* OG1* first opisthosomal ganglion, *OVNC* opisthosomal ventral nerve cord,* PcNv* pectinal nerves,* PeNv 1–4* pedal nerves for legs I–IV,* PpNv* pedipalpal nerves,* SbM* subesophageal mass,* VS* vascular system. Axis indicator abbreviations:* A* anterior,* De* dextral,* P* posterior,* S* sinistral. Scale bars = 0.5 mm. 3D-renderings available in MorphoSource (https://www.morphosource.org; Project ID: 000592858; DOIs: 10.17602/M2/M593464; 10.17602/M2/M592973; 10.17602/M2/M592970; 10.17602/M2/M592967; 10.17602/M2/M593461; 10.17602/M2/M592964).

While the position of the arcuate body and optic neuropils could be recognized or at least estimated in *C. sculpturatus*, *P. becki* and *P. spinigerus* (Figs. 3, 4 and 5), all other internal structures in the brain could only be found in *P. becki* (Vaejovidae) due to its optimal scan quality (Fig. 3). The protocerebral bridge is located just ventro-anteriorly to the arcuate body and connects the two halves of the protocerebrum (Figs. 3, S1). The mushroom body bridge interconnects the two mushroom body calyxes, which are each situated dorsally to their respective peduncli that branch postero-ventrally towards the arcuate body (Fig. 3), and at least two mushroom body lobes could be identified. The globuli cell layer and calyx microglomeruli of the mushroom bodies could not be discerned. The optic neuropil arrangement inside the protocerebrum consists of the first-order median eye neuropil positioned dorso-anteriorly, followed by three neuropils situated in close proximity to each other: the first- and second-order lateral eye neuropils, and the second-order median eye neuropil. The second-order neuropils of the median and lateral eyes are partly merged. A tract, which is only partly visible in our scan, exists interconnecting the two median eye neuropils to the arcuate body (Fig. 3B, C). The arcuate body is positioned dorso-posteriorly in the protocerebrum and its four lobes were only visible in sagittal view. The stomodeal bridge arches anteriorly over the esophagus, extending ventrally on both sides towards the subesophageal mass. However, its connection to the cheliceral neuropils could not be observed. The cheliceral neuropils (deutocerebrum) are situated in an anterior position in both circumesophageal commissures and extend dorso-anteriorly.

### Prosomal ganglion: subesophageal mass

Like the brain, most structures in the subesophageal mass could only be identified in *P. becki*. The paired pedipalpal neuropils (tritocerebrum) are positioned anteriorly in the subesophageal mass, ventral to the cheliceral neuropils (deutocerebrum) of the brain (Fig. 3). Posterior to the paired pedipalpal neuropils, are four pairs of pedal neuropils from which large pedal nerves arise, innervating legs I–IV. The paired central neuropils are located mid-ventrally, extending roughly between the first and third pedal neuropils. Two pairs of pectinal neuropils sit in the posterior portion of the subesophageal mass. The anterior pair, which extends ventro-posteriorly from the central neuropils between the fourth pair of pedal neuropils, is more slender than the posterior pair. Additional subdivisions of the posterior pectinal neuropils, and the accessory and lateral mechanosensory pectinal neuropils as described by Drozd et al.^11,32^ were not observed. Two pairs of major longitudinal fibre tracts, dorso-lateral and ventro-lateral, could be identified in *P. becki*. Additionally, two more pairs of major longitudinal fibre tracts could be located mid-dorsally within the subesophageal mass, however, these two pairs could not be unequivocally assigned as the remaining three pairs could not be found. Likewise, other connecting tracts and the ventral association center were not distinctive enough to observe in the subesophageal mass. Only the large transverse commissures interconnecting the pair of pedipalpal and each pair of pedal neuropils could be identified and no others were found (Figs. 3  and S1).

### Prosomal ganglionic nerves

In all studied specimens, one pair of median eye nerves, one pair of lateral eye nerves and one pair of cheliceral nerves were observed arising from the brain in the prosomal ganglion (Figs. 4 and 5). A pair of lateral nerves was also observed in *H. arizonensis* (Hadruridae) and *P. spinigerus* (Vaejovidae), seemingly arising from the posterior part of the brain (Figs. 4A, D and 5A, D). No other smaller nerves, including accessory cheliceral, accessory, intestinal or rostral nerves (Table S2) could be located confidently. Similarly, in the subesophageal mass of the prosomal ganglion, larger nerves including a pair of pedipalpal nerves, four pairs of pedal nerves, and a pair of pectinal nerves were located in all taxa. One pair of accessory pedipalpal nerves and one pair of dorsal pedal nerves for each leg (I–IV) were only observed in *H. arizonensis* (Hadruridae), however, all other smaller nerves (aortic arch, ephemeral, esophageal, genital, third and fourth mesosomatic segmental, lateral pedal, posterior nerves) could not be located in any specimen. The trajectories of identified nerves were followed until they either became ambiguous due to scan quality or reached their area of innervation.

### Brain size

Brain size was explored, and sizes of the brain, prosomal ganglion and prosoma varied across taxa (Table S5). Brain and prosomal ganglion length were generally positively correlated, with brain length increasing with prosomal ganglion length. Prosomal ganglion and prosomal volume appeared to be inversely proportional, and as prosomal volume decreased, prosomal ganglion volume increased. Prosomal ganglion volume: prosomal volume ratios were highest for *L. australasiae* (1%) followed by *P. becki* (0.7%), *C. sculpturatus* (0.4%), and *H. arizonensis*, *U. mordax* and *P. spinigerus* (0.2%). However, variation in fixation times could have affected this data.

### Opisthosomal ventral nerve cord

Although not the focus of this study, the opisthosomal ventral nerve cord was traced as far as possible in all taxa. The first ganglion (OG1) of the opisthosomal ventral nerve cord was observed in most specimens (Figs. 4 and 5) except *C. sculpturatus*, where only the prosoma and mesosomal segment I were scanned (Figs. 4F and 5F, Table S4), and *U. mordax*, where a motion artifact prevented confident observation. However, the position of OG1 varied in the mesosomal segments of the opisthosoma (O1–O8) across other taxa and was positioned at the genital pore (O2) in *H. arizonensis*; between the genital pore and pectinal base (O2–O3) in *P. becki* and *P. spinigerus*; between the pectinal base and sternite I (O3–O4) in *U. mordax*; and anteriorly in sternite I (O4) in *L. australasiae*. All other free opisthosomal ganglia could be located in the full body scan of *P. becki*: OG2 between book lungs I and II (O4–O5); OG3 at sternite V (O8); OG4 at metasomal segment I (O9); OG5 at metasomal segment II (O10); OG6 at metasomal segment III (O11); and OG7 at metasomal segment IV (O12).

## Discussion

### History of scorpion CNS research and comparison with other arachnids

Prior to the 1960s, studies on the scorpion CNS focused on characterizing its structure in various taxa (Table S1). Newport^33^ and Dufour^34^ dissected several scorpion species and examined CNS gross morphology. Saint-Remy^35–38^ discussed the different regions of the brain and its composition, including the optic and cheliceral neuropils. Laurie^39,40^, Patten^41^, Brauer^42,43^, McClendon^44^ and Abd-el-Wahab^45^ investigated CNS development in scorpion embryos. Lankester^28,29^ reported variation in the positions of opisthosomal ventral nerve cord ganglia and this was further explored by Laurie^30^. Police^46–49^, Hilton^50^, Werner^51^ and Kästner^52^ described internal brain anatomy, different cell types, the subesophageal mass and opisthosomal ventral nerve cord. Several authors including Patten^41^, Haller^53^, Holmgren^54^, Buxton^23^, Hanström^24,55^ and Millot & Vachon^31^ compared the scorpion CNS with other arachnids and with Xiphosura, proposing structural homologies across taxa, although Haller’s^53^ work was heavily criticized by Holmgren^54^ and Gottlieb^56^. Henry^57^ and Ch’eng Pin^58^ detailed nerve innervation patterns in *U. mordax* and the buthid *Mesobuthus tamulus* (Fabricius, 1798), respectively. Morphological research on the scorpion CNS culminated in Babu^5^, who synthesized previous literature and provided comprehensive descriptions and illustrations of the CNS in *Chersonesometrus madraspatensis* (Pocock, 1900) in Scorpionidae Kraepelin, 1905 (Table S1) including the mushroom bodies, arcuate body, opisthosomal ventral nerve cord ganglia and nerve cells. He also compared the scorpion CNS with that of other arachnid orders (Amblypygi, Uropygi, Araneae and Solifugae). Since the 1960s, scorpion CNS research shifted towards physiology^59–85^, with some exceptions^86–90^, and it was not until the current century that interest in CNS morphology resurfaced^11,16,18,25,27,91–97^. Horn and Achaval^91^ found two undescribed pairs of nerves while investigating the CNS of *Bothriurus bonariensis* (C.L. Koch, 1842). In their study of the capillary system surrounding the scorpion prosomal ganglion, Wirkner & Prendini^92^ and Klußmann-Fricke et al.^16,27^ provided 3D-renderings of the scorpion prosomal ganglion. Wolf & Harzsch^25^, Wolf^93,94^ and Drozd et al.^11,32^ studied the pectinal neuropils and their pathways, and Wolf & Harzsch^95,96^ analyzed pedal neuropils. Lehmann & Melzer^18^ reconstructed the optical arrangement in the protocerebrum of the European scorpion genus, *Euscorpius* Thorell, 1876 in Euscorpiidae Laurie, 1896, using both microCT and histology. Wolff & Strausfeld^97^ examined gross morphology of scorpion mushroom bodies with immunohistochemistry.

The present contribution applies microCT to investigate the scorpion CNS. Although scan quality varied across taxa, we visualized all major structures using this technique and our results corroborate previous research. Compared to other arachnids, the scorpion CNS is the least compact^5,23,52^ with the opisthosomal ventral nerve cord constituting seven free ganglia, all of which could be observed in our full body scan of *P. becki* (Fig. 3A). This extended layout most closely resembles that of Xiphosura (horseshoe crabs), which have eight opisthosomal ganglia, including four free anterior ganglia and four fused posterior ganglia^98^. The number of opisthosomal ganglia in other arachnids is far fewer and their position variable, with four spaced ganglia in Palpigradi^99^, one anteriorly positioned and poorly developed ganglion in Solifugae^5,26^, and one posteriorly positioned ganglion in Uropygi^5,26^. In Araneae^5,26^, Acariformes/Parasitiformes^100^, Opiliones^26,101^, Pseudoscorpiones^102^, Ricinulei^103,104^ and Amblypygi^5,26^, no free ganglia are present in the opisthosoma and fused opisthosomal ganglia have been pushed into the subesophageal mass of the prosoma.

The scorpion prosomal ganglion also differs from other arachnids. In *P. becki*, the first-order neuropils of the median and lateral eyes are positioned away from the rest of the brain, whereas the second-order neuropils are adjacent (Fig. 3B–D)—a configuration characteristic to chelicerates^1^. The second-order neuropils of the median and lateral eyes are partly fused with each other in *P. becki* (Fig. 3B–D), and this optical arrangement, in which long fibres of the median and lateral eye neuropils overlap, has already been described for scorpions^18^, Xiphosura^18^, Uropygi^105^ and Amblypygi^106^. This pattern contrasts, however, with Araneae where no overlapping occurs^105^, and with Opiliones/Solifugae^107,108^ and Pseudoscorpiones^109^, which only possess neuropils of the median or lateral eyes, respectively. The arcuate body, present in Xiphosura and all other arachnid orders except Palpigradi^99^ in which it is absent, and Acariformes/Parasitiformes where it absent or vestigial^110^, was located in *P. becki*. It comprises four lobes, the most common number reported for scorpions^5^ (see next section), whereas lobe count for other orders is as follows: four in Uropygi^5,26^, three in Amblypygi^5,26^, two in Solifugae^5,7^ and Pseudoscorpiones^102^, and two to three in Araneae^5,6^. Paired mushroom bodies, centers for memory and sensory integration in arthropods^111^, although their function in scorpions is unknown, were located in *P. becki*, but relatively small in size (0.00084mm^3^), comprising only 0.5% of the prosomal ganglion. Mushroom bodies are also proportionally small in Solifugae^5^, Uropygi^5^ and Pseudoscorpiones^102^, but enormous in Amblypygi^17^ and Xiphosura^111^, and absent in some Araneae^26,112^. The number of mushroom body lobes varies across arachnids with one or two lobes (excluding the pedunculus) in Araneae^6^ and Solifugae^7^, respectively; two or three lobes in Pseudoscorpiones^102^ (although it is unclear whether this count includes the pedunculus); and multiple lobes in Amblypygi and Uropygi^26^. We confidently identified at least two in *P. becki* arising from the base of the pedunculus, although their boundaries were ambiguous and lobe number may vary across scorpions (see below). A mushroom body bridge was observed in *P. becki* and this structure has also been found in Araneae^6^, but its presence in other arachnids is less certain; it appears to be absent in Solifugae^7^ and possibly Pseudoscorpiones^102^. The number of fused ganglia in the subesophageal mass also differs across arachnids, with nine to ten reported in scorpions^23,24^, ten in Solifugae, twelve in Uropygi, sixteen in Araneae and seventeen in Amblypygi^5,26^.

### Variability in the scorpion prosomal ganglion

Previous neuroanatomical studies have stressed that the scorpion CNS is relatively conserved across taxa^5,11,15,25,91^. For example, Wolf & Harzsch^25^ and Drozd et al.^11^ found pectinal neuropils and tracts to be similar across scorpions from different families. Horn & Achaval^91^ identified homologous CNS structures across species. Although we only visualized the prosomal ganglion of *P. becki* in detail, our results corroborate the literature as we found few differences in gross morphology between the prosomal ganglion of *P. becki* and other species previously investigated. Nearly all neuropils (mushroom body, arcuate body, optic, cheliceral, pedipalpal, pectinal, pedal, central), bridges (protocerebral, mushroom body, stomodeal) and major longitudinal fibre tracts (dorso-lateral, ventro-lateral) identified in the brain and subesophageal mass of *C. madraspatensis*^5^ were located in similar positions in *P. becki*. Structures that appeared to be absent (e.g., some fibre tracts, ventral association center) were likely absent due to scan quality. Nerve morphology also seemed conserved across families. Paired optic, cheliceral, pedipalpal, pedal and pectinal nerves were located in all examined taxa, and in all cases, pedipalpal nerves were largest agreeing with previous work^15,91^.

Despite the overarching morphological resemblance of the prosomal ganglion across scorpion taxa, variability has been documented in its component structures^5,24^. For example, optic neuropil counts differ across species^5^ with five median and three lateral eye neuropils reported in the scorpionid *C. madraspatensis*^5^; two median and three lateral eye neuropils in the vaejovid *Paruroctonus boreus* (Girard, 1854)^24^; and four optic neuropils in the buthids *Buthus occitanus* (Amoreux, 1789)^37^ and *Tityus pusillus* Pocock, 1893^54^—the same number observed in our *P. becki* specimen. Lehmann & Melzer^18^ argued that *P. boreus* has four (instead of five) optic neuropils on the basis that Hanström^24^ only recognized the first-order median eye neuropil, mistaking the second-order median eye neuropil as a third-order lateral eye neuropil. However, in his figure, Hanström^24^  labeled the second-order median eye neuropil, considering it fused with the third-order lateral eye neuropil (Hanström^24^: fig. 9), so it is unclear whether Lehmann & Melzer’s^18^ homology assessment is correct. Lobe counts for the scorpion mushroom and arcuate bodies also vary. A single mushroom body lobe was observed in *C. madraspatensis*^5^, whereas three to four lobes were observed in *T. pusillus*^54^, and two in our *P. becki*. We identified four arcuate body lobes in *P. becki*, including two main lobes (an upper and lower with the upper lobe divided into three), agreeing with that observed in *C. madraspatensis*^5^, *Euscorpius*^51,53,56^ and some other scorpion species^52,55^ (Table S1). However, five (three stacked lobes with the upper lobe subdivided into three)^52,55^ and six (one upper and one lower lobe, each divided into three)^54^ lobes have been reported in other species. Further differences have been documented in the subesophageal mass^11,24,26,32^. Nine paired neuropils (ganglia) were reported in the subesophageal mass of *Euscorpius*^42,43^ and *C. madraspatensis*^5^, versus ten in *P. boreus* since Hanström^24^ considered OG1 part of the subesophageal mass due to its close position. We only located eight (one pair of pedipalpal, four pairs of pedal, two pairs of pectinal, one pair of central) in *P. becki*. Drozd et al.^11,32^ highlighted variability in the pectinal neuropils. For example, *Heterometrus* Ehrenberg, 1828 possesses a single accessory pectinal neuropil, whereas *Mesobuthus* Vachon, 1950 has two^11^.

Disparities exist in the literature regarding the smaller nerves stemming from the prosomal ganglion. For many of these nerves, it is not entirely clear where they originate or which regions they innervate, causing confusion. Henry^57^, Babu^5^ and Horn & Achaval^91^ provided detailed diagrams of the nerve innervation pattern in *U. mordax*, *C. madraspatensis* and *B. bonariensis*, respectively, applying different terminologies, sometimes for the same nerve, in each of their studies. Horn & Achaval^91^ noted at least two smaller nerves (paired esophageal and paired aortic arch nerves) in *B. bonariensis*, which could not be assigned to nerves previously described in *U. mordax* and *C. madraspatensis* based on positional differences, and was also doubtful about the homology of several other nerves (e.g., lateral pedal nerves, accessory pedipalpal nerves) across taxa. Unfortunately, most small nerves were not visible in our scans, and only one pair of accessory pedipalpal nerves and four pairs of dorsal pedal nerves originating from the subesophageal mass in *H. arizonensis*, and one pair of lateral nerves originating from the posterior part of the brain in *P. spinigerus* and *H. arizonensis* could be observed. Interestingly, Police^48^ and Henry^57^ documented the lateral nerve (Table S2) in a similar position in *Euscorpius italicus* (Herbst, 1800), and *U. mordax* as in our specimens, whereas Babu^5^ suggested that this nerve originates from the stomodeal bridge in *C. madraspatensis*. Whether this discrepancy represents positional differences of the same nerve or an incorrect homology assessment remains uncertain. Detailed work is required to unravel the homologies of many small nerves in the prosomal ganglion across scorpions.

Our analysis found differences in the size of the prosomal ganglion across taxa. Haller’s Rule states that brain size is inversely related to body size with smaller taxa having a higher brain volume : body mass ratio than larger taxa^113^. Among arachnids, Haller’s Rule has already been documented in Araneae with tiny orb-weaving spiders having brains that overflow into the coxae and comprise up to 78% of the prosomal volume^113^. Our measurement data hints at Haller’s Rule in scorpions with brain volume generally decreasing with increasing prosomal volume as our three largest specimens *H. arizonensis*, *P. spinigerus* and *U. mordax*, had smaller prosomal ganglion: prosomal volume ratios (0.2%) than our three smallest specimens (0.4–1%), *C. sculpturatus*, *P. becki* and *L. australasiae* (Table S5). However, shrinkage artifacts are possible in our dataset and the inclusion of more size-heterogeneous taxa is needed to test this. These results contrast with Drozd et al.^11^ who noted a positive correlation between CNS size and body size, and found proportionally larger anterior pectinal neuropils in bigger (*Heterometrus*) versus smaller (*Mesobuthus*) scorpions. Moreover, sexual dimorphism has been observed in the posterior pectinal neuropils, which comprise 30% versus 10–15% of the subesophageal mass’ width in males and females/juveniles, respectively^93^.

We found brain shape varies across taxonomic groups. In transverse view (Fig. 2), the brains of *P. becki* and *P. spinigerus* in the superfamily Vaejovoidea, and *L. australasiae* in the superfamily Scorpionoidea, are crescent-shaped (Fig. 2A–C). According to phylogenomic data^114^, Vaejovoidea and Scorpionoidea are sister taxa in the parvorder Iurida. Their brain shape contrasts with more distantly related taxa such as the enigmatic *U. mordax* (Fig. 2E), in which the brain appears rectangular (Fig. 2E), and with *C. sculpturatus* in the parvorder Buthida, where the brain appears trapezoidal (Fig. 2F). Our results also allude to a correlation between prosomal ganglion shape and prosomal morphology. The angle between the brain and subesophageal mass in *L. australasiae*, a widespread Asian species that typically resides under tree bark and in rock cracks^115^, was considerably small (< 45° in Fig. 1C), and the subesophageal mass was elongated, conforming to its flattened body shape. We also note a shape difference between the arcuate body of our *P. becki* (Fig. 3B) which is more triangular, and that of *Euscorpius*, which is more curved^18^. Taxonomic differences in the shape of CNS structures have been observed previously. For example, Drozd et al.^11,32^ noted that the anterior pectinal neuropils were ellipsoid in *Heterometrus* and *Euscorpius* (Iurida), whereas they were elongate in *Mesobuthus* (Buthida). Drozd et al.^11,32^ suggested that pectinal neuropil morphology may be correlated with habitat preference since *Heterometrus* and *Euscorpius* are forest-dwelling species, while *Mesobuthus* occurs in dry habitats. *P. becki*, like *Mesobuthus eupeus* (C.L. Koch, 1839), is also dry-adapted, and the anterior pectinal neuropils of the *P. becki* examined here were more elongate, matching the condition in *M. eupeus*. However, whether pectinal neuropil morphology correlates with ecology or evolutionary history has yet to be tested in a phylogenetic framework.

### Opisthosomal ventral nerve cord

The positions of the first three opisthosomal ganglia (OG1–OG3) within the mesosomal segments of the opisthosoma (O1–O8) vary across scorpions and this was previously considered to be an important character for scorpion systematics. Lankester^29^ divided Scorpiones into two subfamilies, Scorpionini and Androctonini, and based his classification in part on the arrangement of opisthosomal ganglia. In his Scorpionini, which included a mixture of Iurida species from several families (Table S1), the prosomal ganglion innervates the first pair of book lungs and opisthosomal ganglia are positioned as follows: OG1 is situated in O4 and innervates the second book lung pair; OG2 is in O6 and innervates the third book lung pair; and OG3 is in O8 and innervates the fourth book lung pair. This contrasts with the pattern observed in his Androctonini, which included only buthids, where the prosomal ganglion innervates both the first and second book lung pairs and opisthosomal ganglia are positioned as such: OG1 lies in O5 and innervates the third book lung pair; OG2 lies in O7 and innervates the fourth book lung pair; and OG3 lies in O8 (last mesosomal segment) and innervates the muscles in the same segment^28,29^. Subsequently, Laurie^30^ emphasized the importance of internal characters in scorpion systematics and re-evaluated positional differences in opisthosomal ventral nerve cord ganglia. He concluded that positions of opisthosomal ganglia were not useful on a higher taxonomic level but could potentially serve to differentiate genera. According to his work, OG1 ranges between O3–O5; OG2 between O6–O7; and OG3 between O6–O8. Laurie^30^ also found, in contrast to Lankester^28,29^, that despite positional differences for OG1–OG3 among species, nerves arising from these ganglia always reached the same location with OG1 innervating the third book lung, OG2 innervating the fourth book lung, and OG3 reaching the muscles in O8 (the last mesosomal segment).

Our work corroborates previous research as the position of OG1 varies among the species studied here (Figs. 4 and 5). We found OG1 to be situated in a more anterior position (O2–O4) than that recorded by Lankester^,28,29^ and Laurie^30^ (O3–O5), and even observed it adjacent to the prosomal ganglion in *P. becki* (Fig. 5). OG1 was illustrated close to the prosomal ganglion in *B. occitanus* (Dufour^34^: fig. 1), and Hanström^55^ even considered OG1 to be incorporated into the prosomal ganglion in *P. boreus*. Like Laurie^30^, we found the position of OG1 to be potentially meaningful at a lower taxonomic level since it was observed in the same position (i.e., between the genital pore and pectinal base) in *P. becki* and *P. spinigerus*, both members of the subfamily Smeringurinae Soleglad & Fet, 2008. However, its significance even at this level remains uncertain since reports vary on whether the opisthosomal ganglia are consistently found in the same positions in a single species. In *Opistophthalmus carinatus* (Peters, 1861), Kästner^52^ observed OG1–OG3 in segments O4, O6 and O8, respectively (Kästner^52^: fig. 105), the same positions noted by Laurie^30^ using a different specimen of the same species. This contrasts with Police^46^, who pointed out that among individuals of *E. italicus*, representing different ages and sizes, OG3 varied between O7–O9.

Regardless of its systematic application, positional variation of the opisthosomal ganglia is perhaps related to ganglion function. The opisthosomal ganglia are subject to spontaneous electrical activity, especially in the mesosoma^61^, and transmit information (in the form of neurosecretions) from the prosomal ganglion to various parts of the opisthosoma. Passing neurosecretions directly from the prosomal ganglion to opisthosomal ganglia seems to help scorpions better coordinate their locomotion with circadian rhythm. The mesosomal ganglia (OG1–OG3), in combination with the subesophageal mass, also provide central control to the scorpion cardiac ganglion situated along the dorsal midline of the heart^116^. Physiological studies suggest that opisthosomal ganglia in the metasoma may be directly sensitive to light^70,74–76^, and have demonstrated different light sensitivities among these ganglia across scorpion species^70^. In *C. madraspatensis*, O5 and O6 were found to be the most sensitive to light, whereas in *Srilankametrus gravimanus* (Pocock, 1894) O7 was the most sensitive. Our understanding of ganglia arrangement in the opisthosomal ventral nerve cord, their function, and their utility in scorpion systematics merits further investigation.

### Limitations of the present work

Our investigation leads to more questions than answers. We recognize the shortcomings of our study, including limited taxon sampling, poor scan resolution and possible shrinkage artifacts. Our analysis included six species representing five families from across the scorpion phylogeny. However, these taxa represent major lineages that are often well-documented in scorpion neuroanatomical research and presently the CNS has been investigated in only 41% (9/22) of scorpion families (Table S1). Characterizing the CNS in more ancient and elusive scorpion families—such as Pseudochactidae Gromov, 1998 or Chaerilidae Pocock, 1893, whose CNS has never been studied before—and optimizing neuroanatomical characters across well-supported trees would help us understand the scorpion CNS in a phylogenetic framework. Scan resolution also varied in our dataset hindering detailed comparison between taxa. We believe that testing different fixation and contrast-enhancing techniques that have been optimized for microCT scanning the CNS in other arachnid orders (e.g., ^6,7,117^) could improve this. We also caution against overinterpretation of the measurement data presented here, which may include shrinkage artifacts. Increasing the sample size per species and standardizing microCT preparation would allow one to test Haller’s rule in scorpions. Finally, we acknowledge that the application of micoCT for understanding the structure and function of the CNS is limited, and future research would benefit by using microCT in combination with other techniques (e.g., histology) to improve results.

## Conclusions

The present contribution investigates the scorpion CNS with microCT and provides the most detailed 3D-rendering of the scorpion prosomal ganglion to date. Our results corroborate existing research and find the scorpion prosomal ganglion to be conserved. Nearly all neuropils, bridges and major fibre tracts reported in previous studies were located in similar positions in *P. becki*, and structures not observed were likely absent due to scan quality. Nerve morphology also appeared to be conserved across families as paired optic, cheliceral, pedipalpal, pedal and pectinal nerves were found in all taxa examined here, and in all cases, pedipalpal nerves were largest. We also report differences from the literature on the prosomal ganglion including the number of mushroom body and arcuate body lobes, number of optic neuropils, shape of arcuate body, and shape and structure of the pectinal neuropils. Variation in brain shape among the taxa studied here may be, in part, phylogenetically informative, and measurement data hint at an inverse relationship between the prosomal ganglion and prosomal volume. The position of OG1 also differed across studied specimens and confirms positional variation in mesosomal ganglia. We recognize the shortcomings of our study, including limited taxon sampling, poor scan resolution, and possible shrinkage artifacts, and believe that applying different techniques could improve comparative findings. In summary, this study is the first to apply microCT to examine morphological variation in the scorpion CNS, and serves as a reference point for future comparative work of this system.

## Materials and methods

### Taxon selection, sample preparation and microCT scanning

Taxa were selected to capture diversity across the order and included six species representing six genera in five families and four superfamilies: *C. sculpturatus* in Buthidae; *H. arizonensis* in Hadruridae; *L. australasiae* in Hormuridae; *P. spinigerus* and *P. becki* in Vaejovidae; and *U. mordax* in Chactidae (see Table S1 for our placement of *U. mordax* in Chactidae). We adopted an exemplar approach and a single specimen of each species was obtained via collecting or through the pet trade and deposited in the California Academy of Sciences (Table S3). Specimens were prepared for microCT scanning considering previous studies on arthropod neuroanatomy or metazoan soft tissues^6,117–120^. Specimens were killed by placing in a freezer for 20 min. Legs and other segments (according to body size) were removed to expedite fixation and dehydration (Table S4). For *P. becki*, a full body scan was performed but later the metasoma was also removed to bring the prosoma closer to the microCT scanner target; however, for all other specimens, only the prosoma and part of the mesosoma were scanned (Table S4). Specimens were submerged in Bouin’s solution (Sigma Aldrich HT10132) with time ranging from a few days to more than one month (Table S4). Following this, specimens were dehydrated in an ascending ethanol series (60%, 60%, 70%, 70%, 80%, 80%, 90%, 90%, 96%, 96%, 100%), allowing between 2 and 3 h for each wash, depending on specimen size, and then placed in 100% ethanol for at least 48 h. Dehydrated specimens were stained in 1% iodine solution, washed again in 100% ethanol and finally critically point dried. MicroCT scanning was performed on a Yxlon FF20 CT scanner (Comet Yxlon, Hamburg) at the California Academy of Sciences. Voltage and current were adjusted for each specimen after trying different scanning parameters to obtain the best contrast and resolution (Table S4).

### 3D-Reconstruction, segmentation and terminology

A CERA reconstruction was performed with the integrated software Reconspooler version 1.2.1.0. After sorting through all reconstructions, only the best scans for each taxon were chosen for segmentation. All segmentations, images, and 3D-renderings were created using VG Studio Max v. 3.5.0 (Volume Graphics, Heidelberg) at the Museum of Nature Hamburg and California Academy of Sciences. The “simple registration” tool was used to acquire equivalent frontal, horizontal and lateral cross-sectional images for each scorpion. A combination of tools, including “Region growing”, “Draw” (with grayscale intervals selected), “Polyline lasso”, “Erode/Dilate”, “Refinement” and “Smoothing” was used to segment the recognizable structures of interest. 3D renderings of each surface were individually colored in the “Rendering” panel. Meshes of all 3D-renderings are deposited in MorphoSource (https://www.morphosource.org; Project ID: 000592858). Measurements of the brain, prosomal ganglion and prosoma were taken in VG Studio Max with the “calipers” tool, and the ratios, *brain length*: *prosomal ganglion length* and *prosomal ganglion volume: prosomal volume* were calculated. We prefer to divide the CNS into ‘prosomal’ and ‘opisthosomal’ regions in order to orient readers across the scorpion bauplan. We adopt the term ‘prosomal ganglion’ from Klußmann-Fricke^27^ to refer to the scorpion’s highly compressed CNS in the prosoma. We replace the terms, ‘opisthosomal ganglion’ and ‘ventral nerve cord’ with a new term ‘opisthosomal ventral nerve cord’ to refer to the scorpion CNS situated in the opisthosoma, given that the CNS in the scorpion opisthosoma comprises a single ventral nerve cord with multiple free ganglia (and their associated nerves) that are not fused into a single ganglion (Table S2). Terminology for other CNS structures are as follows: Richter et al.^10^ for ganglion structure and the protocerebral bridge; Babu^5^ for the longitudinal fibre tracts and ventral association center; Lehmann & Melzer^18^ for the visual system; Richter et al.^10^, Steinhoff et al.^6^ and Wolff & Strausfeld^97^ for mushroom body structure; Loesel et al.^121^ and Strausfeld et al.^122^ for the arcuate body; Horn & Achaval^91^ for the prosomal ganglionic nerves; Brownell^90^, Gaffin^123^, Wolf^94^, Drozd et al.^11^ for pectinal neuropils; Drozd et al.^11^ for use of the term ‘pedal’ instead of ‘leg’ or ‘ambulatory’ neuropils; Steinhoff et al.^6^ for the pedipalpal/cheliceral neuropils and stomodeal bridge; and Sharma et al.^124^ for opisthosomal segmentation numbering (Table S2). We apply current taxonomic identifications when referring to species in past publications (Table S1).

## Electronic supplementary material

Below is the link to the electronic supplementary material.

Supplementary Material 1

## Data Availability

All 3D-renderings illustrated in this study are deposited in MorphoSource (https://www.morphosource.org; Project ID: 000592858; DOIs: 10.17602/M2/M592964; 10.17602/M2/M592967; 10.17602/M2/M592970; 10.17602/M2/M592973; 10.17602/M2/M592979; 10.17602/M2/M592982; 10.17602/M2/M593451; 10.17602/M2/M593461; 10.17602/M2/M593464.
