# Supplementary material for: Revisiting the scorpion central nervous system using microCT
Source: Sci Rep. 2024 Nov 14;14:27961. doi: 10.1038/s41598-024-76917-6 (PMC11564975; doi:10.1038/s41598-024-76917-6)
Supplement: Supplementary file 1 — Supplementary Material 1 [file 41598_2024_76917_MOESM1_ESM.docx]

**Revisiting the Scorpion Central Nervous System Using MicroCT**

Stephanie F. Loria^1,2^*, Valentin L. Ehrenthal^2,3^ and Lauren A. Esposito^1^

^1^Institute for Biodiversity Science and Sustainability, California Academy of Sciences, San Francisco, California, U.S.A.

^2^Museum of Nature Hamburg – Zoology, Leibniz Institute for the Analysis of Biodiversity Change, Hamburg, Germany

^3^Department of Biology, University of Hamburg, Hamburg, Germany

*Corresponding Author: [sloria@calacademy.org](mailto:sloria@calacademy.org); [s.loria@leibniz-lib.de](mailto:s.loria@leibniz-lib.de)

**Table S1.** Taxa previously used in morphological studies on the scorpion central nervous system, including identification based on current taxonomy. Unless contradictory collecting locality information is provided, we follow the original identification and place each species under its currently recognized name.

**Table S2.** Terminology applied in the present paper for the scorpion central nervous system including definitions and synonyms with example references.

**Table S3.** Taxa examined in the present study. All specimens are deposited in the California Academy of Sciences (CASENT).

**Table S4.** Body segments scanned, time in Bouin’s solution (TB), and scanning parameters, including voxel size (VS), voltage (Vt), current (Cu), scan time (ST), integration time (IT) and number of projections (NPr) for specimens used in this study: *Centruroides sculpturatus* Ewing, 1928 in Buthidae C.L. Koch, 1837; *Hadrurus arizonensis* Ewing, 1928 in Hadruridae Stahnke, 1974; *Liocheles australasiae* (Fabricius, 1775) in Hormuridae Laurie, 1896; *Paravaejovis spinigerus* (Wood, 1863) and *Paruroctonus becki* (Gertsch & Allred, 1965) in Vaejovidae Thorell, 1876; and *Uroctonus mordax* Thorell, 1876 in Chactidae Pocock, 1893.

**Table S5.** Measurements of brain length (*Bl*), width (*Bw*) and height (*Bh*); prosomal ganglion volume (*PGv*) and length (*PGl*); prosomal length (*Pl*), anterior width (*PAw*), posterior width (*PPw*), mean width (*PMw* = (*PAw* + *PPw*)/2), height (*Ph*), and volume (*Pv* = *Pl* x *PMw* x *Ph*) for specimens used in this study: *Centruroides sculpturatus* Ewing, 1928 in Buthidae C.L. Koch, 1837; *Hadrurus arizonensis* Ewing, 1928 in Hadruridae Stahnke, 1974; *Liocheles australasiae* (Fabricius, 1775) in Hormuridae Laurie, 1896; *Paravaejovis spinigerus* (Wood, 1863) and *Paruroctonus becki* (Gertsch & Allred, 1965) in Vaejovidae Thorell, 1876; and *Uroctonus mordax* Thorell, 1876 in Chactidae Pocock, 1893. Prosomal length, anterior and posterior width measured along carapace. All measurements were taken in millimeters.

**Fig. S1 Central nervous system of *Paruroctonus becki* (Gertsch & Allred, 1965).** 3D-rendering of the prosomal ganglion of *P. becki* in ventral view. Abbreviations: aPcN: anterior pectinal neuropil; CN: central neuropil; Dlf: dorso-lateral fibre tract; PeN1–PeN4: pedal neuropils for legs I–IV; pPcN: posterior pectinal neuropil; PpN: pedipalpal neuropil; Stb: stomodeal bridge; TC: transverse commissures; Vlf: ventro-lateral fibre tract. Axis indicator abbreviations: A: anterior; De: dextral; P: posterior; S: sinistral. Scale bar = 0.2 mm. 3D-rendering available in MorphoSource (<https://www.morphosource.org>; Project ID: [000592858](https://www.morphosource.org/projects/000592858?locale=en); DOI: [10.17602/M2/M592979](https://doi.org/10.17602/M2/M592979)).

**Supplementary Tables and Figure**

**Table S1.** Taxa previously used in morphological studies on the scorpion central nervous system, including identification based on current taxonomy. Unless contradictory collecting locality information is provided, we follow the original identification and place each species under its currently recognized name.

| **Family** | **Identification** | **Literature Reference** | **Collecting Locality** |
| --- | --- | --- | --- |
| Bothriuridae Simon, 1880 | *Bothriurus bonariensis* (C.L. Koch, 1842) | Horn & Achaval (2002): *Bothriurus bonariensis* (C.L. Koch, 1842) | **Brazil:** Porto Alegre and Barra do Ribeiro |
| Bothriuridae Simon, 1880 | *Bothriurus bonariensis* (C.L. Koch, 1842) | Laurie (1896): *Bothriurus bonariensis* (C.L. Koch, 1842) | No information |
| Bothriuridae Simon, 1880 | *Cercophonius squama* (Gervais, 1843) | Lankester (1885): *Telegonus* sp. (Scorpionini) [This species was referred to as *Cercophonius squama* (Gervais, 1843) in Laurie (1896).] | **Australia:** Tasmania |
| Buthidae C.L. Koch, 1837 | ?*Buthus* sp. | Newport (1843): *Buthus* sp. | No information |
| Buthidae C.L. Koch, 1837 | *Androctonus australis* (Linnaeus, 1758) | Dufour (1856): *Scorpio australis* Linnaeus, 1758 | **Egypt** |
| Buthidae C.L. Koch, 1837 | *Androctonus australis* (Linnaeus, 1758) | Lankester (1882): *Androctonus* (*Prionurus*) *funestus* Ehrenberg, 1828 [This species was referred to as *Prionurus hector* (C.L. Koch, 1839) in Laurie (1896).] | North Africa |
| Buthidae C.L. Koch, 1837 | *Androctonus australis* (Linnaeus, 1758) | Lankester (1885): *Androctonus* (*Prionurus*) *funestus* Ehrenberg, 1828 | North Africa |
| Buthidae C.L. Koch, 1837 | *Androctonus australis* (Linnaeus, 1758) | Newport (1843): *Androctonus* sp. [This species was referred to as *Androctonus* *funestus* Ehrenberg, 1828 in Lankester (1882).] | No information |
| Buthidae C.L. Koch, 1837 | *Androctonus australis* (Linnaeus, 1758) | Wolf & Harzsch (2012): *Androctonus australis* (Linnaeus, 1758) | **Egypt** |
| Buthidae C.L. Koch, 1837 | *Androctonus australis* (Linnaeus, 1758) | Saint-Remy (1887a): *Androctonus funestus* Ehrenberg, 1828 (*Sc. tunetanus* Redi, *Sc. australis* L.) | No information |
| Buthidae C.L. Koch, 1837 | *Buthacus arenicola* (Simon, 1885) | Wirkner & Prendini (2007): *Buthacus arenicola* (Simon, 1885) | No information |
| Buthidae C.L. Koch, 1837 | *Buthus occitanus* (Amoreux, 1789) | Saint-Remy (1886a, 1886b, 1887a, 1887b): ‘scorpion’/*Buthus occitanus* (Amoreux, 1789) | **France:** Banyuls-sur-Mer |
| Buthidae C.L. Koch, 1837 | *Buthus occitanus* (Amoreux, 1789) | Buxton (1917): *Buthus occitanus* (Amoreux, 1789) | No information |
| Buthidae C.L. Koch, 1837 | *Buthus occitanus* (Amoreux, 1789) | Dufour (1856): *Scorpio europaeus* Linnaeus, 1758 | No information |
| Buthidae C.L. Koch, 1837 | *Buthus occitanus* (Amoreux, 1789) | Dufour (1856): *Scorpio occitanus* Amoreux, 1789 [This species was referred to as *Androctonus occitanus* (Amoreux, 1789) in Lankester (1882), and as *Buthus europaeus* Thorell, 1876 in Laurie (1896).] | **France:** cotes mediterraneennes de France |
| Buthidae C.L. Koch, 1837 | *Buthus occitanus* (Amoreux, 1789) | Lankester (1885): *Androctonus occitanus* (Amoreux, 1789) | No information |
| Buthidae C.L. Koch, 1837 | *Buthus occitanus* (Amoreux, 1789) | Laurie (1896): *Androctonus occitanus* (Amoreux, 1789) | No information |
| Buthidae C.L. Koch, 1837 | *Centruroides gracilis* (Latreille, 1804) | Lankester (1885): *Androctonus americanus* [This species was referred to as *Centrurus gracilis* (Latreille, 1804) in Laurie (1896).] | No information |
| Buthidae C.L. Koch, 1837 | *Centruroides gracilis* (Latreille, 1804). [See Fet & Lowe (2000).] | Dufour (1856): *Scorpio* *biaculeatus* (Lucas, 1835) | No information |
| Buthidae C.L. Koch, 1837 | *Centruroides* sp. | Kästner (1941): *Centruroides* sp. | No information |
| Buthidae C.L. Koch, 1837 | *Centruroides* sp. | Laurie (1896): *Centrurus* sp. | No information |
| Buthidae C.L. Koch, 1837 | *Centruroides vittatus* (Say, 1821) [See Fet & Lowe (2000).] | McClendon (1904): *Buthus carolinianus* | **U.S.A.:** Texas: Austin |
| Buthidae C.L. Koch, 1837 | *Centruroides vittatus* (Say, 1821) [See Fet & Lowe (2000).] | Patten (1890): ?*Buthus carolinianus* | No information |
| Buthidae C.L. Koch, 1837 | *Hottentotta hottentotta* (Fabricius, 1787) | Lankester (1885): *Androctonus hottentotus* (Fabricius, 1787) | No information |
| Buthidae C.L. Koch, 1837 | *Hottentotta tamulus* (Fabricius, 1798). [See Fet & Lowe (2000).] | Dufour (1856): *Scorpio* *nigrolineatus* Dufour, 1856 | No information |
| Buthidae C.L. Koch, 1837 | *Hottentotta tamulus* (Fabricius, 1798). [See Fet & Lowe (2000).] | Laurie (1896): *Buthus nigrolineatus* (Dufour, 1856) | No information |
| Buthidae C.L. Koch, 1837 | *Leiurus quinquestriatus* (Ehrenberg, 1828) | Wolf & Harzsch (2002a): *Leiurus quinquestriatus* (Ehrenberg, 1828) | **Israel:** Judean desert |
| Buthidae C.L. Koch, 1837 | *Leiurus quinquestriatus* (Ehrenberg, 1828) | Wolf & Harzsch (2002b): *Leiurus quinquestriatus* (Ehrenberg, 1828) | **Israel:** Judean desert |
| Buthidae C.L. Koch, 1837 | *Leiurus quinquestriatus* (Ehrenberg, 1828) | Wolf (2008): *Leiurus quinquestriatus* (Ehrenberg, 1828) | **Israel:** Judean desert |
| Buthidae C.L. Koch, 1837 | *Leiurus quinquestriatus* (Ehrenberg, 1828) | Klußmann-Fricke et al. (2012): *Leiurus quinquestriatus* (Ehrenberg, 1828) | No information |
| Buthidae C.L. Koch, 1837 | *Leiurus quinquestriatus* (Hemprich & Ehrenberg, 1828) | Khalil et al. (1985): *Buthus quinquestriatus* (Hemprich & Ehrenberg, 1828) | **Egypt:** Giza: Abu-Roach |
| Buthidae C.L. Koch, 1837 | *Leiurus quinquestriatus* (Hemprich & Ehrenberg, 1928) | Abd-el-Wahab (1952): *Buthus quinquestriatus* (Hemprich & Ehrenberg, 1829) | **Egypt:** Cairo |
| Buthidae C.L. Koch, 1837 | *Mesobuthus eupeus* (C.L. Koch, 1839) | Drozd et al. (2020): *Mesobuthus eupeus* (C.L. Koch, 1839) | Pet trade |
| Buthidae C.L. Koch, 1837 | *Mesobuthus eupeus* (C.L. Koch, 1839) | Drozd et al. (2022): *Mesobuthus eupeus* (C.L. Koch, 1839) | Pet trade |
| Buthidae C.L. Koch, 1837 | *Olivierus martensii* (Karsch, 1879) | Ch’eng (1939): *Buthus martensi* Karsch, 1879 | **China:** Beijing |
| Buthidae C.L. Koch, 1837 | *Parabuthus capensis* (Ehrenberg, 1831) | Kästner (1941): *Parabuthus capensis* (Ehrenberg, 1831) | No information |
| Buthidae C.L. Koch, 1837 | *Parabuthus capensis* (Ehrenberg, 1831) | Laurie (1896): *Parabuthus capensis* (Ehrenberg, 1831) | No information |
| Buthidae C.L. Koch, 1837 | *Parabuthus* sp. | Millot & Vachon (1949): *Parabuthus* sp. | No information |
| Buthidae C.L. Koch, 1837 | *Tityus serrulatus* Lutz & Mello, 1922 | Lucas et al. (1965): *Tityus serrulatus* Lutz & Mello, 1922 | **Brazil:** Minas Gerais: Nova Era |
| Buthidae C.L. Koch, 1837 | *Tityus* sp. [*T. pusillus* is does not occur in Suriname. See Fet & Lowe (2000).] | *Holmgren (1916): *Tityus pusillus* Pocock, 1893 | **Suriname** |
| Buthidae C.L. Koch, 1837 | *Uroplectes triangulifer* (Thorell, 1876) | Laurie (1896): *Uroplectes triangulifer* (Thorell, 1876) | No information |
| Caraboctonidae Kraepelin, 1905 | *Caraboctonus keyserlingi* Pocock, 1893 | Lankester (1885): *Telegonus* sp. (Scorpionini) [This species was referred to as *Caraboctonus keyserlingii* Pocock, 1893 in Laurie (1896).] | **Chile:** Coquimbo |
| Chactidae Pocock, 1893 | *Brotheas granulatus* Simon, 1877 | Klußmann-Fricke et al. (2012): *Brotheas granulatus* Simon, 1877 | No information |
| Chactidae Pocock, 1893 | *Brotheas granulatus* Simon, 1877 | Klußmann-Fricke et al. (2014): *Brotheas granulatus* Simon, 1877 | No information |
| Chactidae Pocock, 1893 | *Neochactas delicatus* (Karsch, 1879) | Laurie (1896): *Broteochactas delicatus* (Karsch, 1879) | No information |
| Chactidae Pocock, 1893 | *Teuthraustes atramentarius* Simon, 1878 | Lankester (1885): *Brotheas subnitens* (Scorpionini) [This species was referred to as *Teuthraustes atramentarius* Simon, 1878 in Laurie (1896).] | No information |
| Chactidae Pocock, 1893 | *Uroctonus mordax* Thorell, 1876 [The familial position of this species is uncertain and it has been placed in both Vaejovidae Thorell, 1876 and Chactidae Pocock, 1893. See Santibáñez‐López et al. (2023) for a discussion on its uncertain phylogenomic position. Given this uncertainty, we follow Soleglad & Fet (2003) and leave this species in Chactidae in order to distinguish it from the true Vaejovidae species in our microCT analysis.] | **Henry (1949): *Uroctones mordax* Thorell, 1876 | **U.S.A.:** Mendocino Co. |
| Euscorpiidae Laurie, 1896 | *Euscorpius carpathicus* (Linnaeus, 1767) | Haller (1912): *Scorpio europaeus* Linnaeus, 1758 [After following Kraepelin’s (1899) catalog, Gottlieb (1926) identified Haller’s (1912) *S. europaeus* as conspecific with his *E. carpathicus*.] | No information |
| Euscorpiidae Laurie, 1896 | *Euscorpius carpathicus* (Linnaeus, 1767) | Kästner (1941): *Euscorpius carpathicus* (Linnaeus, 1767) | No information |
| Euscorpiidae Laurie, 1896 | *Euscorpius carpathicus* (Linnaeus, 1767)/*Euscorpius flavicaudis* (DeGeer, 1778) | Saint-Remy (1887a): *Scorpio europaeus* Latr. (*Euscorpius carpathicus* L., *Scorpio flavicaudus* de Geer) | No information |
| Euscorpiidae Laurie, 1896 | *Euscorpius hadzii* Di Caporiacco, 1950 | Lehmann & Melzer (2013): *Euscorpius hadzii* Di Caporiacco, 1950 | Pet trade |
| Euscorpiidae Laurie, 1896 | *Euscorpius italicus* (Herbst, 1800) | Drozd et al. (2022): *Euscorpius italicus* (Herbst, 1800) | **Italy:** just north of Bolzano, foothills of the Dolomitic Alps, 800 m, Sept. 2019 |
| Euscorpiidae Laurie, 1896 | *Euscorpius italicus* (Herbst, 1800) | Kästner (1941): *Euscorpius italicus* (Herbst, 1800) | No information |
| Euscorpiidae Laurie, 1896 | *Euscorpius italicus* (Herbst, 1800) | Lankester (1882): *Scorpio italicus* Herbst, 1800 [This species was referred to as *Euscorpius italicus* (Herbst, 1800) in Laurie (1896).] | No information |
| Euscorpiidae Laurie, 1896 | *Euscorpius italicus* (Herbst, 1800) | Laurie (1890, 1896): *Euscorpius italicus* (Herbst, 1800) | **Italy:** Naples |
| Euscorpiidae Laurie, 1896 | *Euscorpius italicus* (Herbst, 1800) | Lehmann & Melzer (2013): *Euscorpius italicus* (Herbst, 1800) | **Croatia:** Rovinj |
| Euscorpiidae Laurie, 1896 | *Euscorpius italicus* (Herbst, 1800) | Police (1901, 1902, 1903, 1904): *Euscorpius italicus* (Herbst, 1800) | No information |
| Euscorpiidae Laurie, 1896 | *Euscorpius tergestinus* (C.L. Koch, 1837)*/Euscorpius aquilejensis* (C.L. Koch, 1837). [See Tropea (2013).] | Brauer (1894, 1895): *Euscorpius carpathicus* (Linnaeus, 1767) | **Italy:** Trieste |
| Euscorpiidae Laurie, 1896 | *Euscorpius tergestinus* (C.L. Koch, 1837)*/Euscorpius aquilejensis* (C.L. Koch, 1837). [See Tropea (2013).] | Gottlieb (1926): *Euscorpius carpathicus* (Linnaeus, 1767) | **Italy:** Trieste |
| Hadruridae Stahnke, 1974 | *Hadrurus hirsutus* (Wood, 1863) | Wolf & Harzsch (2002a): *Hadrurus hirsutus* (Wood, 1863) | **U.S.A.:** California: near Palm Springs, Mojave Desert |
| Hadruridae Stahnke, 1974 | *Hadrurus hirsutus* (Wood, 1863) | Wolf & Harzsch (2002b): *Hadrurus hirsutus* (Wood, 1863) | **U.S.A.:** California: near Palm Springs, Mojave Desert |
| Hadruridae Stahnke, 1974 | *Hadrurus hirsutus* (Wood, 1863) | Wolf (2008): *Hadrurus hirsutus* (Wood, 1863) | **U.S.A.:** California: near Palm Springs, Mojave Desert |
| Hormuridae Laurie, 1896 | *Liocheles australasiae* (Fabricius, 1775) | Laurie (1896): *Hormurus australasiae* (Fabricius, 1775) | No information |
| Hormuridae Laurie, 1896 | *Opisthacanthus madagascariensis* Kraepelin, 1894 | Laurie (1896): *Opisthocentrus madagascariensis* (Kraepelin, 1894) | No information |
| Hormuridae Laurie, 1896 | *Opisthacanthus validus* Thorell, 1876 | Laurie (1896): *Opisthocentrus validus* (Thorell, 1876) | No information |
| Scorpionidae Kraepelin, 1905 | Unknown | Newport (1843): *Scorpius* sp. | No information |
| Scorpionidae Kraepelin, 1905 | *Chersonesometrus madraspatensis* (Pocock, 1900). [See Prendini & Loria (2020).] | Babu (1965): *Heterometrus fulvipes* (C.L. Koch, 1837) | **India:** Tirupati |
| Scorpionidae Kraepelin, 1905 | *Chersonesometrus madraspatensis* (Pocock, 1900). [See Prendini & Loria (2020).] | Laurie (1890): *Scorpio* (*Buthus*) *fulvipes* (C.L. Koch, 1837);  Laurie (1891, 1896): *Scorpio fulvipes* (C.L. Koch, 1837) | **India:** Madras |
| Scorpionidae Kraepelin, 1905 | *Chersonesometrus madraspatensis* (Pocock, 1900). [See Prendini & Loria (2020).] | Yellamma et al. (1982): *Heterometrus fulvipes* (C.L. Koch, 1837) | **India:** Tirupati |
| Scorpionidae Kraepelin, 1905 | *Gigantometrus swammerdami* (Simon, 1872) | Habibulla (1970): *Heterometrus swammerdami* (Simon, 1872) | **India:** Tirupati |
| Scorpionidae Kraepelin, 1905 | *Heterometrus longimanus* (Herbst, 1800) | Kästner (1941): *Heterometrus longimanus* (Herbst, 1800) | No information |
| Scorpionidae Kraepelin, 1905 | *Heterometrus* sp. | ***Drozd et al. (2020): *Heterometrus petersii* (Thorell, 1876) | Pet trade |
| Scorpionidae Kraepelin, 1905 | *Heterometrus* sp. | ***Wolf & Harzsch (2002a): *Heterometrus petersii* (Thorell, 1876) | Pet trade |
| Scorpionidae Kraepelin, 1905 | *Heterometrus* sp. | ***Wolf & Harzsch (2002b): *Heterometrus petersii* (Thorell, 1876) | Pet trade |
| Scorpionidae Kraepelin, 1905 | *Heterometrus* sp. | ***Wolf (2008): *Heterometrus petersii* (Thorell, 1876) | Pet trade |
| Scorpionidae Kraepelin, 1905 | *Heterometrus thorellii* (Pocock, 1892) | Laurie (1896): *Palamnaeus thorellii* Pocock, 1892 | No information |
| Scorpionidae Kraepelin, 1905 | *Opistophthalmus capensis* (Herbst, 1800) | Laurie (1896): *Opistophthalmus capensis* (Herbst, 1800) | No information |
| Scorpionidae Kraepelin, 1905 | *Opistophthalmus carinatus* (Peters, 1861) | Kästner (1941): *Opistophthalmus carinatus* (Peters, 1861) | No information |
| Scorpionidae Kraepelin, 1905 | *Pandinus imperator* (C.L. Koch, 1841) | Wolf & Harzsch (2012): *Pandinus imperator* (C.L. Koch, 1841) | Pet trade |
| Scorpionidae Kraepelin, 1905 | *Sahyadrimetrus scaber* (Thorell, 1876). [See Prendini & Loria (2020).] | Dufour (1856): *Scorpio leioderma* (Dufour, 1856) | No information |
| Scorpionidae Kraepelin, 1905 | *Scorpio palmatus* (Ehrenberg, 1828) | Dufour (1856): *Scorpio* (*Buthus*) *palmatus* Ehrenberg, 1828 | No information |
| Scorpionidae Kraepelin, 1905 | *Scorpio palmatus* (Ehrenberg, 1828)/*Scorpio maurus* Linnaeus, 1758 | Saint-Remy (1887a): *Heterometrus palmatus* (Ehrenberg, 1828) (*Buthus palmatus* Ehr., *Scorpio maurus* L.) | No information |
| Scorpionidae Kraepelin, 1905 | *Srilankametrus indus* (DeGeer, 1778). [See Prendini & Loria (2020).] | Lankester (1882): *Scorpio cyaneus* (C.L. Koch, 1836) [This species was referred to as *Scorpio megacephalus* (C.L. Koch, 1936) in Laurie (1896).] | **Sri Lanka:** Ceylon |
| Scorpionidae Kraepelin, 1905 | *Srilankametrus indus* (DeGeer, 1778). [See Prendini & Loria (2020).] | Lankester (1885): *Scorpio cyaneus* (C.L. Koch, 1836) (Scorpionini) | **Sri Lanka:** Ceylon |
| Scorpionidae Kraepelin, 1905 | *Srilankametrus indus* (DeGeer, 1778)/*Javanimetrus cyaneus* (C.L. Koch, 1836). [See Prendini & Loria (2020).] | Dufour (1856): *Scorpio afer* Linnaeus, 1758 | No information |
| Scorpionidae Kraepelin, 1905 | Unknown [See Fet (2000).] | Dufour (1856): *Scorpio* *longicauda* Latreille, 1804 | No information |
| Vaejovidae Thorell, 1876 | *Paravaejovis flavus* (Banks, 1900) | Wolf (2008): *Vaejovis flavus* Banks, 1900 | **U.S.A.:** California: near Palm Springs, Mojave Desert |
| Vaejovidae Thorell, 1876 | *Paravaejovis flavus* (Banks, 1900) | Wolf & Harzsch (2002a): *Vaejovis flavus* Banks, 1900 | **U.S.A.:** California: near Palm Springs, Mojave Desert |
| Vaejovidae Thorell, 1876 | *Paravaejovis flavus* (Banks, 1900) | Wolf & Harzsch (2002b): *Vaejovis flavus* Banks, 1900 | **U.S.A.:** California: near Palm Springs, Mojave Desert |
| Vaejovidae Thorell, 1876 | *Paravaejovis spinigerus* (Wood, 1863) | Wolf (2008): *Vaejovis spinigerus* (Wood, 1863) | **U.S.A.:** California: near Palm Springs, Mojave Desert |
| Vaejovidae Thorell, 1876 | *Paravaejovis spinigerus* (Wood, 1863) | Wolf (2017): *Vaejovis spinigerus* (Wood, 1863) | No information |
| Vaejovidae Thorell, 1876 | *Paravaejovis spinigerus* (Wood, 1863) | Wolf & Harzsch (2002a): *Vaejovis spinigerus* (Wood, 1863) | **U.S.A.:** California: near Palm Springs, Mojave Desert |
| Vaejovidae Thorell, 1876 | *Paravaejovis spinigerus* (Wood, 1863) | Wolf & Harzsch (2002b): *Vaejovis spinigerus* (Wood, 1863) | **U.S.A.:** California: near Palm Springs, Mojave Desert |
| Vaejovidae Thorell, 1876 | *Paravaejovis spinigerus* (Wood, 1863) | Wolff & Strausfeld (2015): *Hoffmannius spinigerus* (Wood, 1863) | No information |
| Vaejovidae Thorell, 1876 | *Paruroctonus boreus* (Girard, 1854) | Hanström (1923): *Vejovis boreus* (Girard, 1854) | **U.S.A.:** California: San Diego Co.: Scripps Institute |
| Vaejovidae Thorell, 1876 | *Paruroctonus boreus* (Girard, 1854) | Millot & Vachon (1949): *Vejovis boreus* (Girard, 1854) | No information |
| Vaejovidae Thorell, 1876 | *Smeringurus mesaensis* (Stahnke, 1957) | Brownell (1998): *Paruroctonus mesaensis* Stahnke, 1957 | No information |
| Vaejovidae Thorell, 1876 | *Smeringurus mesaensis* (Stahnke, 1957) | Bowerman & Burrows (1980): *Paruroctonus mesaensis* Stahnke, 1957 | **U.S.A.:** Arizona |
| Vaejovidae Thorell, 1876 | *Smeringurus mesaensis* (Stahnke, 1957) | Lane & Harrison (1980): *Paruroctonus mesaensis* Stahnke, 1957 | No information |
| Vaejovidae Thorell, 1876 | *Vaejovis* sp. | Laurie (1896): *Vejovis* sp. | No information |

*Holmgren (1916) also compared *T. pusillus* with the genera *Buthus* Leach, 1815, *Scorpio* Linnaeus, 1758, *Pandinus* C.L. Koch, 1841 and *Grosphus* Simon, 1880*.* However, no further information was provided on these genera so their identifications cannot be validated.

**Henry (1949) stated that he compared *U. mordax* with *Isometrus maculatus* (DeGeer, 1778) from the Mount Hamilton Range, California. However, no further details were provided on the neuroanatomy of this specimen and all illustrations refer to *U. mordax*.

***Prendini & Loria (2020) updated the classification of Heterometrinae Simon, 1879. *Heterometrus petersii* (Thorell, 1876) from Vietnam was found to be *Heterometrus silenus* (Simon, 1884), whereas *Heterometrus petersii* was determined to be a cryptic species from Penang Island, Malaysia and Singapore. Given that the locality data for this specimen is unknown, it is impossible to know which of these two species it is.

**References**

Abd-el-Wahab A. Some notes on the segmentation of the scorpion, *Buthus quinquestriatius* (H.&E.). Proceedings of the Egyptian Academy of Sciences. 1952;7:75–91.

Babu KS. Anatomy of the central nervous system of arachnids. Zoologische Jahrbucher, Abteilung für Anatomie und Ontogenie. 1965;82:1–154.

Bowerman RF, Burrows M. The morphology and physiology of some walking leg motor neurons in a scorpion. Journal of Comparative Physiology A. 1980;140:31–42. <https://doi.org/10.1007/BF00613745>

Brauer A. Beiträge zur Kenntnis der Entwicklungsgeschichte des Skorpions. I. Zeitschrift für Wissenschaftliche Zoologie. 1894;57:402–432, pl. XIX–XX.

Brauer A. Beiträge zur Kenntnis der Entwicklungsgeschichte des Skorpions. II. Zeitschrift für Wissenschaftliche Zoologie. 1895;59:351–435, pl. XXI–XXV.

Brownell PH. Glomerular Cytoarchitectures in Chemosensory Systems of Arachnids. Annals of the New York Academy of Sciences. 1998;855(1):502–507. <https://doi.org/10.1111/j.1749-6632.1998.tb10614.x>

Buxton BH. Notes on the anatomy of arachnids. Journal of Morphology. 1917;29(1):1–25, pl. I–III.

Ch’eng-Pin P. Morphology and anatomy of the Chinese scorpion *Buthus martensi* Karsch. Peking Natural History Bulletin. 1939;14(2):103–117, pl. I–II.

Drozd D, Wolf H, Stemme T. Structure of the pecten neuropil pathway and its innervation by bimodal peg afferents in two scorpion species. PLoS One. 2020;15(12):e0243753. <https://doi.org/10.1371/journal.pone.0243753>

Drozd D, Wolf H, Stemme T. Mechanosensory pathways of scorpion pecten hair sensillae — Adjustment of body height and pecten position. Journal of Comparative Neurology. 2022;530:2918–2937. <https://doi.org/10.1002/cne.25384>

Dufour L. Histoire anatomique et physiologique des scorpions. Mémoires Présentés par Divers Savants à l'Académie des Sciences de l’Institut Impérial de France. 1856;14;561–653.

Fet V, Lowe G. Family Buthidae C.L. Koch, 1837. In: Fet V, Sissom WD, Lowe G, Braunwalder ME, editors. Catalog of the scorpions of the World (1758–1998). New York: The New York Entomological Society; 2000. p. 54–286.

Fet V. Family Scorpionidae Latreille, 1802. In: Fet V, Sissom WD, Lowe G, Braunwalder ME, editors. Catalog of the scorpions of the World (1758–1998). New York: The New York Entomological Society; 2000. p. 427–486.

Gottlieb K. Über das Gehirn des Skorpions. Zeitschrift für wissenschaftliche Zoologie. 1926;127:185–243, pl. III–IV.

Habibulla M. Neurosecretion in the scorpion *Heterometrus swammerdami*. Journal of Morphology. 1970;131:1–16. <https://doi.org/10.1002/jmor.1051310102>

Haller B. Über das Zentralnervensystem des Skorpions und der Spinnen. Archiv für mikroskopische Anatomie. 1912;79(1):504–524, pl. XXVI.

Hanström B. Further notes on the central nervous system of arachnids: Scorpions, phalangids, and trap‐door spiders. Journal of Comparative Neurology. 1923;35(4):249–274.

Henry L. The nervous system and the segmentation of the head in the scorpion (Arachnida). Microentomology. 1949;14(4):121–126.

Holmgren N. Zur vergleichenden Anatomie des Gehirns: von Polychaeten. Onychophoren, Xiphosuren, Arachniden, Crustaceen, Myriapoden und Insekten. Vorstudien zu einer Phylogenie der Arthropoden. Kungliga Svenska Vetenskapsakademiens Handlingar. Stockholm. 1916;56(1):1–303, pl. I–XII.

Horn ACM, Achaval M. The gross anatomy of the nervous system of *Bothriurus bonariensis* (L.C. Koch, 1842) (Scorpiones, Bothriuridae). Brazilian Journal of Biology. 2002;62:253–262. <https://doi.org/10.1590/S1519-69842002000200009>

Kästner A. 1. Ordnung der Arachnida: Scorpiones. In: Krumbach T, editor. Handbuch der Zoologie. Berlin: Walter de Gruyter Verlag; 1941;3:117–240.

Khalil A, Ismail S, El-Bakary Z. Studies on the morphology and histology of the central nervous system of the adult of the Egyptian scorpion *Buthus quinquestriatus* (H.E.) II – General Histology. Assiut Veterinary Medical Journal. 1985;14(28):75–83. <https://doi.org/10.21608/avmj.1985.190235>

Klußmann-Fricke BJ, Pomrehn SW, Wirkner CS. A wonderful network unraveled – Detailed description of capillaries in the prosomal ganglion of scorpions. Frontiers in Zoology. 2014;11:1–5. <https://doi.org/10.1186/1742-9994-11-28>

Klußmann-Fricke BJ, Prendini L, Wirkner CS. Evolutionary morphology of the hemolymph vascular system in scorpions: A character analysis. Arthropod Structure & Development. 2012;41(6):545–560. <https://doi.org/10.1016/j.asd.2012.06.002>

Kraepelin K, Scorpiones und Pedipalpi. In: Dahl F, editor. Das Tierreich. Berlin: Friedländer und Sohn Verlag, Herausgegeben von der Deutschen Zoologischen Gesellschaft; 1899; 8(Arachnoidea):1–265.

Lane NJ, Harrison JB. An unusual form of tight junction in the nervous-system of the scorpion. European Journal of Cell Biology. 1980;22:244–244.

Lankester ER. Part V. Notes on certain points in the anatomy and generic characters of scorpions. Transactions of the Zoological Society of London. 1885;11:372–384, pl. LXXXII–LXXXIII.

Lankester ER. VI. Note on the difference in the position of the ganglia of the ventral nerve-cord in three species of scorpion. Proceedings of the Royal Society of London. 1882;34(220):101–104. <https://doi.org/10.1098/rspl.1882.0019>

Laurie M. Notes on the anatomy of some scorpions, and its bearing on the classification of the order. Annals and Magazine of Natural History (Ser. 6). 1896;17:185–193.

Laurie M. Some points in the development of *Scorpio fulvipes*. Quarterly Journal of Microscopical Science, New Ser. 1891;32:587–597, pl. XL. <https://doi.org/10.1242/jcs.s2-32.128.587>

Laurie M. The embryology of the scorpion (*Euscorpius italicus*). Quarterly Journal of Microscopical Science, New Ser. 1890;31:105–141, pl. XIII–XVIII. <https://doi.org/10.1242/jcs.s2-31.122.105>

Lehmann T, Melzer RR. Looking like *Limulus*? – Retinula axons and visual neuropils of the median and lateral eyes of scorpions. Frontiers in Zoology. 2013;10:40. <https://doi.org/10.1186/1742-9994-10-40>

Lucas S, v. Eichstedt VD, Bucherl W. Sobre o Sistema nervosa de *Tityus serrulatus* – Titynae – Buthidae – Scorpiones. Memórias do Instituto de Butantan. 1965;32:15–26.

McClendon JF. On the anatomy and embryology of the nervous system of the scorpion. The Biological Bulletin. 1904;8(1):38–55.

Millot J, Vachon M. Ordre des scorpions. In: Grasse P-P, editor. Traité de zoologie. Paris: Masson et Cie; 1949; 6: 386–436.

Newport G. VIII. On the structure, relations, and development of the nervous and circulatory systems, and on the existence of a complete circulation of the blood in vessels, in Myriapoda and macrourous Arachnida.—First series. Philosophical Transactions of the Royal Society of London. 1843;(133):243–302. <https://doi.org/10.1098/rstl.1843.0013>

Patten W. Memoirs: On the origin of vertebrates from arachnids. Journal of Cell Science. 1890;2(123):317–378. <https://doi.org/10.1242/jcs.s2-31.123.317>

Police G. Ricerche sul Sistema nervosa dell’ *Euscorpius italicus*. Atti della Reale Accademia delle Scienze Fisiche e Matematiche – Società Reale di Napoli. ser. 2. 1901;10(7):1–10, figs. 1–4.

Police G. Sui centri nervosi dei cheliceri e del rostro nello skorpione. Bolletino della Società di Naturalisti Napoli*.* ser. 1. 1904;18:130–135.

Police G. Sui centri nervosi sottointestinali dell' *Euscorpius italicus*. Bolletino della Società di Naturalisti Napoli. ser. 1. 1902;15:1*–*24, figs. 1–12.

Police G. Sul sistema nervoso stomatogastrico dello scorpione. Archivio Zoologico: Pubblicato sotto gli auspicii della unione zoologica italiana. 1903;1:179–200, figs. 1–10.

Prendini L, Loria SF. Systematic revision of the Asian forest scorpions (Heterometrinae Simon, 1879), revised suprageneric classification of Scorpionidae Latreille, 1802, and revalidation of Rugodentidae Bastawade et al., 2005. Bulletin of the American Museum of Natural History. 2020;442(1):1–480. <https://doi.org/10.1206/0003-0090.442.1.1>

Saint-Remy G. Recherches sur la structure du cerveau du scorpion. Comptes Rendus de l’Académie des Sciences. 1886a;102:1492–1494.

Saint-Remy G. Structure des centres nerveux chez le scorpion. Bulletin de la Société des Sciences de Nancy. 1886b;(2)8(20):xxix.

Saint-Remy G. Contribution à l'étude du cerveau chez les Arthropodes trachéates. Archives de Zoologie Expérimentale et Génerale. Ser. 2 Supp. 1887a;5(2):1–274.

Saint-Remy G. Structure du cerveau chez le scorpion et la scolopendre. Bulletin de la Société des Sciences de Nancy. 1887b;(2)9(21):xxxi–xxxii.

Santibáñez‐López CE, Ojanguren‐Affilastro AA, Graham MR, Sharma PP. Congruence between ultraconserved element‐based matrices and phylotranscriptomic datasets in the scorpion Tree of Life. Cladistics. 2023;39(6):533-47. <https://doi.org/10.1111/cla.12551>

Soleglad ME, Fet V. High-level systematics and phylogeny of the extant scorpions (Scorpiones: Orthosterni). Euscorpius. 2003;11:1–57. <https://dx.doi.org/10.18590/euscorpius.2003.vol2003.iss11.1>

Tropea G. Reconsideration of the taxonomy of *Euscorpius tergestinus* (Scorpiones: Euscorpiidae). Euscorpius. 2013;162:1–23. <https://dx.doi.org/10.18590/euscorpius.2013.vol2013.iss162.1>

Wirkner CS, Prendini L. Comparative morphology of the hemolymph vascular system in scorpions – A survey using corrosion casting, MicroCT, and 3D‐reconstruction. Journal of Morphology. 2007;268(5):401–413. <https://doi.org/10.1002/jmor.10512>

Wolf H. The pectine organs of the scorpion, *Vaejovis spinigerus*: Structure and (glomerular) central projections. Arthropod Structure & Development. 2008;37(1):67–80. <https://doi.org/10.1016/j.asd.2007.05.003>

Wolf H. Scorpions pectines – Idiosyncratic chemo-and mechanosensory organs. Arthropod Structure & Development. 2017;46(6):753–764. <https://doi.org/10.1016/j.asd.2017.10.002>

Wolf H, Harzsch S. Evolution of the arthropod neuromuscular system. 1. Arrangement of muscles and innervation in the walking legs of a scorpion: *Vaejovis spinigerus* (Wood, 1863) Vaejovidae, Scorpiones, Arachnida. Arthropod Structure & Development. 2002a;31(3):185–202. <https://doi.org/10.1016/S1467-8039(02)00043-9>

Wolf H, Harzsch S. Evolution of the arthropod neuromuscular system. 2. Inhibitory innervation of the walking legs of a scorpion: *Vaejovis spinigerus* (Wood, 1863), Vaejovidae, Scorpiones, Arachnida. Arthropod Structure & Development. 2002b;31(3):203–215. <https://doi.org/10.1016/S1467-8039(02)00044-0>

Wolf H, Harzsch S. Serotonin-immunoreactive neurons in scorpion pectine neuropils: Similarities to insect and crustacean primary olfactory centres? Zoology. 2012;115(3):151–159. <https://doi.org/10.1016/j.zool.2011.10.002>

Wolff GH, Strausfeld NJ. Genealogical correspondence of mushroom bodies across invertebrate phyla. Current Biology. 2015;25(1):38–44. <https://doi.org/10.1016/j.cub.2014.10.049>

Yellamma K, Subhashini K, Murali Mohan P, Babu KS. Microanatomy of the 7th abdominal ganglion and its peripheral nerves in the scorpion, *Heterometrus fulvipes*. Proceedings: Animal Sciences. 1982;91(3);225–234. <https://doi.org/10.1007/BF03185013>

**Table S2.** Terminology applied in the present paper for the scorpion central nervous system including definitions and synonyms with example references.

| **Preferred Term** | **Definition** | **Synonyms** |
| --- | --- | --- |
| *Accessory cheliceral nerves* (1 pair) | Small nerves innervating the chelicerae. | *2P nerves* (McClendon 1904); *small cheliceral nerve* (Henry 1949: ventro-retrolateral pair); *AC1*: *accessory nerves 1* (Babu 1965). See Horn & Achaval (2002) for discussion on the homology of these nerves. |
| *Accessory nerves* (1 pair) | Small nerves in the region of the cheliceral nerves, innervating an unknown region. | *4A nerve* (McClendon 1904); *cheliceral nerve* (Henry 1949: dorso-internal pair); *AC2*: *accessory nerve 2* (Babu 1965). See Horn & Achaval (2002) for discussion on the homology of these nerves. |
| *Accessory pectinal neuropils* (1 or 2 pairs) | Smaller neuropils adjacent to the anterior pectinal neuropils. Not documented in all species. See Drozd et al. (2020, 2022). |  |
| *Accessory pedipalpal nerves* (1 pair) | Small nerves innervating the pedipalps. See Horn & Achaval (2002). | *small nerves from basal region of pedipalps* (Babu 1965). See Horn & Achaval (2002) for discussion on the homology of these nerves. |
| *Anterior pectinal neuropils* (1 pair) | The anterior pair of pectinal neuropils. | *anterior pecten neuropil* (Drozd et al. 2020, 2022); *anterior pectine neuropil* (Wolf 2008) |
| *Aortic arch nerves* (1 pair) | Nerves innervating aortic arches. See Horn & Achaval (2002). |  |
| *Arcuate body* | See Loesel et al. (2002) and Strausfeld et al. (2006). | *central body* (Babu 1965); *striped body* (Babu 1965) |
| *Brain* | Includes the protocerebrum (including arcuate body, optic neuropils and mushroom bodies) + deutocerebrum (including cheliceral neuropil) and associated connectives (e.g., protocerebral bridge, mushroom body bridge, etc.). | *supra-oesophageal ganglion* (Ch’eng-Pin 1939); *supraesophageal ganglion* (Horn & Achaval 2002; Klußmann-Fricke et al. 2012); *supraesophageal ganglionic mass*; *cerebral ganglion* (Henry 1949); *cerebral nerve mass*; *syncerebrum* |
| *Central major longitudinal fibre tracts* (1 pair) | Longitudinal fibre tracts in the subesophageal mass positioned centrally. See Babu (1965). | ?*central longitudinal sensory tracts* (Drozd et al. 2022) |
| *Central nervous system* | Prosomal ganglion + opisthosomal ventral nerve cord. |  |
| *Central neuropils* (1 pair) | Neuropils located mid-ventrally between first and third pedal neuropils. See Babu (1965). | *central ganglion* (Babu 1965); *central tract* (Wolf & Harzsch 2002) |
| *Centro-lateral major longitudinal fibre tracts* (1 pair) | Longitudinal fibre tracts in the subesophageal mass positioned centro-laterally. See Babu (1965). |  |
| *Cheliceral nerves* (1 pair) | Nerves innervating the chelicerae. See Horn & Achaval (2002). |  |
| *Cheliceral neuropils* (1 pair) | Neuropils of the chelicerae in the deutocerebrum. See Steinhoff et al. (2017). |  |
| *Circumesophageal commissures* | These connect the brain situated dorsally and subesophageal mass situated ventrally. |  |
| *Deutocerebrum* | Includes the paired cheliceral neuropils. |  |
| *Dorsal pedal nerves* (4 pairs) | Small nerves situated dorsally above pedal nerves. See Horn & Achaval (2002) about uncertain innervation region. | ?*amnd1–4*: *ambulatory dorsal nerves 1–4* (Babu 1965); *dorsal ambulatory nerves* (Horn & Achaval 2002). See Horn & Achaval (2002) for discussion on the questionable homology of these nerves. |
| *Dorso-lateral major longitudinal fibre tracts* (1 pair) | Longitudinal fibre tracts in the subesophageal mass positioned dorso-laterally. See Babu (1965). | ?*dorsal longitudinal sensory tracts* (Drozd et al. 2022) |
| *Ephemeral nerves* (1 pair) | Small nerves potentially innervating dorsal muscles in prosoma and endosternite. See Horn & Achaval (2002) on questionable innervation region. | ?*integument nerves* (Henry 1949) |
| *Esophageal nerves* (1 pair) | Nerves innervating the esophagus. See Horn & Achaval (2002). |  |
| *Fourth mesosomatic segmental nerves* | Small nerves innervating the fourth segment of the mesosoma. See Horn & Achaval (2002). | *11P* (McClendon 1904); *4abdn*: *4^th^ mesosomatic segmental nerve* (Babu 1965); *9* (Lucas et al. 1965: fig. 1); *vagus nerves* (Hjelle 1990). See Horn & Achaval (2002) for discussion on the homology of these nerves. |
| *Genital nerves* (2 pairs) | Two pairs of small nerves (one originating dorsally, the other ventrally) uniting and later forking to the genital pore and surrounding region. See Horn & Achaval (2002). | ?*operculum*/*gonad/gonopore nerves* (Henry 1949); *Gnd*: *anterior genital nerve* + *Gnv*: *small posterior genital nerve* (Babu 1965). See Horn & Achaval (2002) for discussion on the homology of these nerves. |
| *Intestinal nerves* (1 pair) | Nerves originating from stomodeal bridge and innervating anterior part of the intestines. See Gottlieb (1926), Henry (1949) and Babu (1965). | *stomatogastric nerves* (Police 1903); ?*recurrent nerve* (Henry 1949) |
| *Lateral eye nerves* (1 pair) | Nerves innervating the lateral eyes. |  |
| *Lateral eye neuropils* | Neuropils of the lateral eyes. |  |
| *Lateral mechanosensory neuropils* | Small neuropils near the posterior pectinal neuropil. See Drozd et al. (2022). |  |
| *Lateral nerves* (1 pair) | Nerves originating from posterior part of the brain. Police (1903) suggests this nerve originates from the arcuate body and Henry (1949) places this nerve in a similar position. However, Babu (1965) suggests this nerve originates from the stomodeal bridge. We follow Babu (1965) and assume these nerves are homologous until further evidence is given. | ?*tegumentary nerve* (Henry 1949) |
| *Lateral pedal nerves* (5 pairs) | Small nerves innervating legs I–IV: one nerve to legs I, III and IV; two nerves for leg II. See Horn & Achaval (2002). | *small nerves from basal region of legs* (Babu 1965); *pedal accessory nerves* (Horn & Achaval 2002). See Horn & Achaval (2002) for discussion on the homology of these nerves. |
| *Longitudinal fibre tracts* (7 pairs) | Fibre tracts located within the subesophageal mass that extend posteriorly into the opisthosomal ventral nerve cord. Includes seven major pairs: central tract, centro-lateral, dorso-lateral, mid-central, mid-dorsal, mid-ventral, ventro-lateral fibre tracts. See Babu (1965). |  |
| *Median eye nerves* (1 pair) | Nerves innervating the median eyes. |  |
| *Median eye neuropils* | Neuropils of the median eyes. |  |
| *Mesosomal ganglia* (4) | Includes four mesosomal ganglia (including pectinal, genital pore and book lung ganglia) incorporated into the subesophageal mass. | *Posterior neuropils* (Klußmann-Fricke et al. 2014) |
| *Mid-central major longitudinal fibre tracts* (1 pair) | Longitudinal fibre tracts in the subesophageal mass positioned mid-centrally. See Babu (1965). |  |
| *Mid-dorsal major longitudinal fibre tracts* (1 pair) | Longitudinal fibre tracts in the subesophageal mass positioned mid-dorsally. See Babu (1965). |  |
| *Mid-ventral major longitudinal fibre tracts* (1 pair) | Longitudinal fibre tracts in the subesophageal mass positioned mid-ventrally. See Babu (1965). |  |
| *Mushroom body* (1 pair) | Includes a calyx + pedunculus + lobes. See Richter et al. (2010). | *corpora pedunculata* (Babu 1965); *glomeruli* (Babu 1965) |
| *Mushroom body bridge* | Connecting the mushroom body lobes anteriorly. | *anterior protocerebral bridge* (Babu 1965) |
| *Mushroom body calyx* | See Richter et al. (2010). |  |
| *Mushroom body globuli cell layer* | Globuli layer surrounding mushroom body calyx. See Strausfeld et al. (2020) and Sinakevitch et al. (2021). |  |
| *Mushroom body microglomeruli* | Region of the mushroom body calyx. See Sinakevitch et al. (2021). |  |
| *Mushroom body pedunculus* | See Richter et al. (2010). |  |
| *Neurilemma* | Sheath surrounding the brain and subesophageal mass. See Richter et al. (2010). |  |
| *Opisthosomal ganglia* (7) | Seven ganglia including three in the mesosoma (OG1–OG3), and four in the metasoma (OG4–OG7). |  |
| *Opisthosomal ventral nerve cord* | Ventral nerve cord comprising 3 mesosomal + 4 metasomal ganglia and associated nerves in the opisthosoma. | *opisthosoma ganglion*; *ventral nerve cord* (Horn & Achaval 2002) |
| *Optic neuropils* | Neuropils of the median and lateral eyes. |  |
| *Pectinal nerves* (1 pair) | Nerves innervating the pectines. See Horn & Achaval (2002). | *pecten nerves* (Henry 1949) |
| *Pectinal neuropils* | Neuropils of the pectines, two major pairs (anterior and posterior pectinal neuropils) and several minor (e.g., lateral mechanosensory and accessory pectinal neuropils). See Brownell (1998), Gaffin (2002) and Drozd et al. (2020, 2022) for information and further subdivisions. | *pectinal masses* (Babu 1965) |
| *Pedal nerves* (4 pairs for legs I–IV) | Nerves innervating legs I–IV. | *crural nerves* (Ch’eng-Pin 1939; Henry 1949) |
| *Pedal neuropils* (4 pairs for legs I–IV) | Neuropils of legs I–IV. See Drozd et al. (2020). |  |
| *Pedipalpal nerves* (1 pair) | Nerves innervating the pedipalps. See Horn & Achaval (2002). |  |
| *Pedipalpal neuropils* (1 pair) | Neuropils of the pedipalp in the tritocerebrum. See Steinhoff et al. (2017). |  |
| *Posterior nerves* (1 pair) | Nerves innervating the abdomen in unknown region. See Horn & Achaval (2002). | ?*10A*: (McClendon 1904). See Horn & Achaval (2002) for discussion on the homology of these nerves. |
| *Posterior pectinal neuropils* (1 pair) | Posterior pair of pectinal neuropils that can be further subdivided. See Drozd et al. (2020, 2022). |  |
| *Prosomal ganglion* | Brain + subesophageal mass + circumesophageal commissures + associated nerves. See Klußmann-Fricke et al. (2012). | *cephalothoracic mass* (Babu 1965); *cephalic ganglionic mass* (Lane & Harrison 1980); *synganglion* (Steinhoff et al. 2017) |
| *Protocerebral bridge* | Connecting the two halves of protocerebrum. See Richter et al. (2010). | *posterior protocerebral bridge* (Babu 1965) |
| *Protocerebrum* | Includes optic neuropils + mushroom bodies + arcuate body. |  |
| *Rostral nerves* (unpaired) | Stemming anteriorly from stomodeal bridge, innervating the rostrum. See Horn & Achaval (2002). | *labrum nerve* (Henry 1949) |
| *Stomodeal bridge* | Connecting the cheliceral neuropils, arching anteriorly over esophagus. See Steinhoff et al. (2017). | *frontal ganglion* (Henry 1949); *sympathetic rostral ganglion* (Babu 1965). See Bitsch & Bitsch (2007). |
| *Subesophageal mass* | Refers to the part of the CNS that is ventral to the brain in the scorpion prosomal ganglion. The subesophageal mass consists of multiple fused ganglia with their component neuropils (see Richter et al. 2010: fig. 9 for structure of a ganglion). The number of fused ganglia in the subesophageal mass varies in the literature but the following have been identified: one ganglion of the pedipalps; four pedal ganglia for legs I–IV; four mesosomal ganglia (including the pectinal, genital pore and book lungs) that have been incorporated into the subesophageal mass; and the central ganglion. The subesophageal mass also includes longitudinal fibre and other connecting tracts, transverse commissures and the ventral association center. | *subo-esophageal ganglion* (Buxton 1917; Ch’eng-Pin 1939; Henry 1949); *subesophageal ganglion* (Klußmann-Fricke et al. 2012, 2014); *thoracic nerve mass*; *subesophageal ganglionic mass*; *hindbrain* (Hjelle 1990) |
| *Third mesosomatic segmental nerves* (1 pair) | Small nerves innervating the third segment of the mesosoma. See Horn & Achaval (2002). | *11A* (McClendon 1904); *3abdn*: *3^rd^ mesosomatic segmental nerve* (Babu 1965); *8* (Lucas et al. 1965: fig. 1); *vagus nerves* (Hjelle 1990). See Horn & Achaval (2002) for discussion on the homology of these nerves. |
| *Transverse commissures* | Commissures connecting neuropils bilaterally. |  |
| *Tritocerebrum* | Includes the pedipalpal neuropils. |  |
| *Ventral association center* | Ventral region of subesophageal mass. See Babu (1965). |  |
| *Ventral connectives* (1 pair) | Large nerves connecting subesophageal mass to first opisthosomal ganglion of opisthosomal ventral nerve cord. See Ch’eng-Pin (1939), Babu (1965) and Hjelle (1990). | *connectives* (Horn & Achaval 2002) |
| *Ventro-lateral major longitudinal fibre tracts* (1 pair) | Longitudinal fibre tracts in the subesophageal mass positioned ventro-laterally. See Babu (1965). | ?*ventral longitudinal sensory tract* (Drozd et al. 2022) |

**References**

Babu KS. Anatomy of the central nervous system of arachnids. Zoologische Jahrbucher, Abteilung für Anatomie und Ontogenie. 1965;82:1–154.

Bitsch J, Bitsch C. The segmental organization of the head region in Chelicerata: a critical review of recent studies and hypotheses. Acta Zool. 88(4), 317–335. <https://doi.org/10.1111/j.1463-6395.2007.00284.x>

Brownell PH. Glomerular Cytoarchitectures in Chemosensory Systems of Arachnids. Annals of the New York Academy of Sciences. 1998;855(1):502–507. <https://doi.org/10.1111/j.1749-6632.1998.tb10614.x>

Buxton BH. Notes on the anatomy of arachnids. Journal of Morphology. 1917;29(1):1–25, pl. I–III.

Ch’eng-Pin P. Morphology and anatomy of the Chinese scorpion *Buthus martensi* Karsch. Peking Natural History Bulletin. 1939;14(2):103–117, pl. I–II.

Drozd D, Wolf H, Stemme T. Structure of the pecten neuropil pathway and its innervation by bimodal peg afferents in two scorpion species. PLoS One. 2020;15(12):e0243753. <https://doi.org/10.1371/journal.pone.0243753>

Drozd D, Wolf H, Stemme T. Mechanosensory pathways of scorpion pecten hair sensillae — Adjustment of body height and pecten position. Journal of Comparative Neurology. 2022;530:2918–2937. <https://doi.org/10.1002/cne.25384>

Gaffin DD. Electrophysiological analysis of synaptic interactions within peg sensilla of scorpion pectines. Microscopy Research and Technique. 2002;58(4):325–334. <https://doi.org/10.1002/jemt.10140>

Gottlieb K. Über das Gehirn des Skorpions. Zeitschrift für wissenschaftliche Zoologie. 1926;127:185–243, pl. III–IV.

Henry L. The nervous system and the segmentation of the head in the scorpion (Arachnida). Microentomology. 1949;14(4):121–126.

Hjelle JT. Anatomy and morphology. In: Polis GA, editor. The biology of scorpions. Stanford, CA: Stanford University Press; 1990. p. 9–63.

Horn ACM, Achaval M. The gross anatomy of the nervous system of *Bothriurus bonariensis* (L.C. Koch, 1842) (Scorpiones, Bothriuridae). Brazilian Journal of Biology. 2002;62:253–262. <https://doi.org/10.1590/S1519-69842002000200009>

Klußmann-Fricke BJ, Prendini L, Wirkner CS. Evolutionary morphology of the hemolymph vascular system in scorpions: A character analysis. Arthropod Structure & Development. 2012;41(6):545–560. <https://doi.org/10.1016/j.asd.2012.06.002>

Klußmann-Fricke BJ, Pomrehn SW, Wirkner CS. A wonderful network unraveled – Detailed description of capillaries in the prosomal ganglion of scorpions. Frontiers in Zoology. 2014;11:1–5. <https://doi.org/10.1186/1742-9994-11-28>

Lane NJ, Harrison JB. An unusual form of tight junction in the nervous-system of the scorpion. European Journal of Cell Biology. 1980;22:244–244.

Loesel R, Nässel DR, Strausfeld NJ. Common design in a unique midline neuropil in the brains of arthropods. Arthropod Structure & Development. 2002;31(1):77–91. <https://doi.org/10.1016/S1467-8039(02)00017-8>

Lucas S, v. Eichstedt VD, Bucherl W. Sobre o Sistema nervosa de *Tityus serrulatus* – Titynae – Buthidae – Scorpiones. Memórias do Instituto de Butantan. 1965;32:15–26.

McClendon JF. On the anatomy and embryology of the nervous system of the scorpion. The Biological Bulletin. 1904;8(1):38–55.

Police G. Sul sistema nervoso stomatogastrico dello scorpione. Archivio Zoologico: Pubblicato sotto gli auspicii della unione zoologica italiana. 1903;1:179–200, figs. 1–10.

Richter S, Loesel R, Purschke G, Schmidt-Rhaesa A, Scholtz G, Stach T, Vogt L, Wanninger A, Brenneis G, Döring C, Faller S. Invertebrate neurophylogeny: Suggested terms and definitions for a neuroanatomical glossary. Frontiers in Zoology. 2010;7:29. <https://doi.org/10.1186/1742-9994-7-29>

Sinakevitch I, Long SM, Gronenberg W. The central nervous system of whip spiders (Amblypygi): Large mushroom bodies receive olfactory and visual input. Journal of Comparative Neurology. 2021;529(7):1642–1658. <https://doi.org/10.1002/cne.25045>

Steinhoff PO, Sombke A, Liedtke J, Schneider JM, Harzsch S, Uhl G. The synganglion of the jumping spider *Marpissa muscosa* (Arachnida: Salticidae): Insights from histology, immunohistochemistry and microCT analysis. Arthropod Structure & Development. 2017;46(2):156–170. <https://doi.org/10.1016/j.asd.2016.11.003>

Strausfeld NJ, Strausfeld C, Stowe S, Rowell D, Loesel R. Arthropod phylogeny: Onychophoran brain organization suggests an archaic relationship with a chelicerate stem lineage. Proceedings of the Royal Society B: Biological Sciences. 2006;273:1857–1866. <https://doi.org/10.1098/rspb.2006.3536>

Strausfeld NJ, Wolff GH, Sayre ME. Mushroom body evolution demonstrates homology and divergence across Pancrustacea. eLife. 2020;9:e52411. <https://doi.org/10.7554/eLife.52411>

Wolf H. The pectine organs of the scorpion, *Vaejovis spinigerus*: Structure and (glomerular) central projections. Arthropod Structure & Development. 2008;37(1):67–80. <https://doi.org/10.1016/j.asd.2007.05.003>

Wolf H, Harzsch S. Evolution of the arthropod neuromuscular system. 1. Arrangement of muscles and innervation in the walking legs of a scorpion: *Vaejovis spinigerus* (Wood, 1863) Vaejovidae, Scorpiones, Arachnida. Arthropod Structure & Development. 2002;31(3):185–202. <https://doi.org/10.1016/S1467-8039(02)00043-9>

**Table S3.** Taxa examined in the present study. All specimens are deposited in the California Academy of Sciences (CASENT).

| **Superfamily** | **Family** | **Species** | **Collecting Locality** |
| --- | --- | --- | --- |
| Buthoidea | Buthidae C.L. Koch, 1837 | *Centruroides* *sculpturatus* Ewing, 1928 | **U.S.A.:** Arizona: Maricopa County: First Water Road, Trailhead Horse Lot, Apache Junction, 33.48718 -111.444404, L.A. Esposito, (CASENT 9101993) |
| Hadruroidea | Hadruridae Stahnke, 1974 | *Hadrurus arizonensis* Ewing, 1928 | **U.S.A.:** Arizona, 2017, (CASENT 9101994) |
| Scorpionoidea | Hormuridae Laurie, 1896 | *Liocheles australasiae* (Fabricius, 1775) | **Malaysia:** Penang: Penang Hill, 2017 Penang Hill Bioblitz, 16–27.x.2017, L.A. Esposito & S.F. Loria, (CASENT 9101995) |
| Vaejovoidea | Vaejovidae Thorell, 1876 |  |  |
|  | Smeringurinae Soleglad & Fet, 2008 | *Paravaejovis spinigerus* (Wood, 1863) | **U.S.A.:** Arizona: Pinal County: Hieroglyphic Trailhead, Gold Canyon, 33.391495 -111.423385, L.A. Esposito, (CASENT 9101996) |
|  | Smeringurinae Soleglad & Fet, 2008 | *Paruroctonus becki* (Gertsch & Allred, 1965) | **U.S.A.:** California, (CASENT 9101997) |
| Incertae sedis | *Chactidae Pocock, 1893 | *Uroctonus mordax* Thorell, 1876 | **U.S.A.:** California: Marin County: Mill Valley, Troop 80 Trail, 37°54'32.2''N 122°35'24.6''W, 309 m, 19. Aug. 2017, S.F. Loria, (CASENT 9101998) |

*See Table S1 regarding our placement of *U. mordax* in Chactidae.

**Table S4.** Body segments fixed and scanned, time in Bouin’s solution (TB), and scanning parameters, including voxel size (VS), voltage (Vt), current (Cu), scan time (ST), integration time (IT) and number of projections (NPr) for specimens used in this study: *Centruroides sculpturatus* Ewing, 1928 in Buthidae C.L. Koch, 1837; *Hadrurus arizonensis* Ewing, 1928 in Hadruridae Stahnke, 1974; *Liocheles australasiae* (Fabricius, 1775) in Hormuridae Laurie, 1896; *Paravaejovis spinigerus* (Wood, 1863) and *Paruroctonus becki* (Gertsch & Allred, 1965) in Vaejovidae Thorell, 1876; and *Uroctonus mordax* Thorell, 1876 in Chactidae Pocock, 1893. Segment abbreviations are as follows: P: Prosoma; Pe: Pedipalps; Ms: Mesosoma; Mt: Metasoma; Fe: Femur.

| **Species** | **Fixed**  **Segments** | **TB (days)** | **Scanned Segments** | **VS (µm)** | **Vt**  **(kV)** | **Cu (µA)** | **ST (min)** | **IT**  **(µs)** | **NPr** |
| --- | --- | --- | --- | --- | --- | --- | --- | --- | --- |
| *C. sculpturatus* | P + Pe (Fe only) + Ms + Mt (I, II only) | 30 | P + Ms (I only) | 3.88 | 40 | 705 | 96 | 2000000 | 1440 |
| *H. arizonensis* | P + Ms (I, II only) | 3 | P + Ms (I only) | 10.87 | 40 | 700 | 144 | 2000000 | 1440 |
| *L. australasiae* | P + Pe + Ms + Mt | 3 | P + Ms (I, II only) | 3.36 | 40 | 700 | 144 | 2000000 | 1440 |
| *P. becki* | P + Pe + Ms + Mt | 2 | P + Ms (I, II only) | 3.87 | 40 | 700 | 288 | 2000000 | 1440 |
|  |  |  | P + Pe + Ms + Mt | 50.07 | 70 | 65 | 28 | 333333 | 1440 |
| *P. spinigerus* | P + Ms + Mt (I, II only) | 30 | P + Ms (I, II only) | 6.74 | 40 | 600 | 144 | 2000000 | 1440 |
| *U. mordax* | P + Ms | 3 | P + Ms (I only) | 6.73 | 40 | 700 | 144 | 2000000 | 1440 |

**Table S5.** Measurements of brain length (*Bl*), width (*Bw*) and height (*Bh*); prosomal ganglion volume (*PGv*) and length (*PGl*); prosomal length (*Pl*), anterior width (*PAw*), posterior width (*PPw*), mean width (*PMw* = (*PAw* + *PPw*)/2), height (*Ph*), and volume (*Pv* = *Pl* x *PMw* x *Ph*) for specimens used in this study: *Centruroides sculpturatus* Ewing, 1928 in Buthidae C.L. Koch, 1837; *Hadrurus arizonensis* Ewing, 1928 in Hadruridae Stahnke, 1974; *Liocheles australasiae* (Fabricius, 1775) in Hormuridae Laurie, 1896; *Paravaejovis spinigerus* (Wood, 1863) and *Paruroctonus becki* (Gertsch & Allred, 1965) in Vaejovidae Thorell, 1876; and *Uroctonus mordax* Thorell, 1876 in Chactidae Pocock, 1893. Prosomal length, anterior and posterior width measured along carapace. All measurements were taken in millimeters.

| **Value** | ***C. sculpturatus*** | ****H. arizonensis*** | ***L. australasiae*** | ***P. spinigerus*** | ***P. becki*** | ***U. mordax*** |
| --- | --- | --- | --- | --- | --- | --- |
| *Bl* | 0.42 | 1.03 | 0.36 | 0.49 | 0.44 | 0.67 |
| *Bw* | 0.55 | 1.57 | 0.52 | 0.64 | 0.76 | 1.06 |
| *Bh* | 0.35 | 0.41 | 0.29 | 0.34 | 0.40 | 0.32 |
| *PGv* | 0.19 | 2.00 | 0.10 | 0.29 | 0.17 | 0.32 |
| *PGl* | 1.62 | 2.51 | 1.12 | 1.48 | 1.35 | 1.98 |
| *Pl* | 4.81 | 12.21 | 3.99 | 7.28 | 3.83 | 7.41 |
| *PAw* | 2.96 | 7.67 | 2.28 | 4.67 | 2.32 | 4.46 |
| *PPw* | 4.62 | 11.70 | 4.18 | 7.23 | 3.33 | 7.39 |
| *PMw* | 3.79 | 9.69 | 3.23 | 5.95 | 2.83 | 5.93 |
| *Ph* | 2.76 | 7.63 | 0.83 | 4.50 | 2.12 | 4.10 |
| *Pv* | 50.31 | 902.74 | 10.70 | 194.92 | 22.98 | 180.16 |
| *Bl*: *PGl* | 0.2593 | 0.4104 | 0.3214 | 0.3311 | 0.3259 | 0.3384 |
| *PGv*: *Pv* | 0.0037 | 0.0022 | 0.0098 | 0.0015 | 0.0074 | 0.0018 |

*A large air gap was observed within the brain of *H. arizonensis*, suggesting damage occurred during sample preparation so measurements for this specimen should be read with caution.

**Fig. S1 Central nervous system of *Paruroctonus becki* (Gertsch & Allred, 1965).** 3D-rendering of the prosomal ganglion of *P. becki* in ventral view. Abbreviations: aPcN: anterior pectinal neuropil; CN: central neuropil; Dlf: dorso-lateral fibre tract; PeN1–PeN4: pedal neuropils for legs I–IV; pPcN: posterior pectinal neuropil; PpN: pedipalpal neuropil; Stb: stomodeal bridge; TC: transverse commissures; Vlf: ventro-lateral fibre tract. Axis indicator abbreviations: A: anterior; De: dextral; P: posterior; S: sinistral. Scale bar = 0.2 mm. 3D-rendering available in MorphoSource (<https://www.morphosource.org>; Project ID: [000592858](https://www.morphosource.org/projects/000592858?locale=en); DOI: [10.17602/M2/M592979](https://doi.org/10.17602/M2/M592979)).
